# Quantification and description of photothermal heating effects in plasmon-assisted electrochemistry

**DOI:** 10.1038/s42004-024-01157-8

**Published:** 2024-04-01

**Authors:** Md. Al-Amin, Johann V. Hemmer, Padmanabh B. Joshi, Kimber Fogelman, Andrew J. Wilson

**Affiliations:** 1 Department of Chemistry, University of Louisville, Louisville, KY 40292 USA; 2 Present Address: Duke University, Durham, NC 27708 USA

**Keywords:** Physical chemistry, Electrochemistry, Nanoparticles, Photocatalysis, Energy transfer

## Abstract

A growing number of reports have demonstrated plasmon-assisted electrochemical reactions, though debate exists around the mechanisms underlying the enhanced activity. Here we address the impact of plasmonic photothermal heating with cyclic voltammetry measurements and finite-element simulations. We find that plasmonic photothermal heating causes a reduction in the hysteresis of the anodic and cathodic waves of the voltammograms along with an increase in mass-transport limiting current density due to convection induced by a temperature gradient. At slow scan rates, a temperature difference as low as 1 K between the electrode surface and bulk electrolytic solution enhances the current density greater than 100%. Direct interband excitation of Au exclusively enhances current density by photothermal heating, while plasmon excitation leads to photothermal and nonthermal enhancements. Our study reveals the role of temperature gradients in plasmon-assisted electrochemistry and details a simple control experiment to account for photothermal heating.

## Abstract (web summary)

Highly energetic charge carriers generated in plasmon-assisted electrocatalysis can increase reaction rates and impart novel selectivity trends, but a simple protocol to differentiate between thermal and nonthermal plasmonic contributions is lacking. Here, the authors use cyclic voltammetry and finite element simulations to show direct interband excitation of gold by visible light exclusively enhances current density via photothermal heating, while plasmon excitation leads to photothermal and nonthermal enhancements.

## Introduction

Utilizing the effects of localized surface plasmon excitation and decay in electrochemistry has garnered increasing interest recently. Excitation of plasmon resonances leads to concentration of electromagnetic fields, generation of hot charge carriers, electrostatic potentials, and localized heating, all of which have the potential to affect electrochemical reactions. Further, the materials that have visible light plasmon resonances are coinage metals, which are also active catalysts for many important electrocatalytic transformations. Utilizing the hot (highly energetic) charge carriers generated in plasmon-assisted catalysis is driving significant interest due to the potential to increase reaction rates and impart novel selectivity trends. Also of interest is that chemical transformations on plasmonic electrode materials can utilize both the hot electrons and hot holes, a distinct advantage over semiconductor photoelectrodes which typically serve as either photoanodes or photocathodes (i.e., n-type or p-type). Despite low quantum efficiencies for charge carrier extraction in plasmonic materials, nontrivial enhancements to reaction rates and apparent modulation in reaction selectivity have been reported^1–7^.

Several aspects of plasmon excitation and decay have been cited in the mechanisms of light-enhanced electrochemical reactions. The strong electromagnetic fields in gaps between plasmonic nanostructures have been postulated to assist electrochemical reactions through an optical rectification process^8–10^, where a dc electric potential is established from the ac electromagnetic field. This photopotential has been described as a supplement to the applied electrical bias in electrochemical cells. Excitation off the plasmon resonance peak has also been shown to establish surface potentials which may contribute to driving electrochemical reactions^11–13^. Hot charge carriers have the potential to contribute directly to redox reactions or may assist in the vibrational activation of adsorbates^14,15^. It has also been suggested that photoexcited charges can accumulate on plasmonic metals if there is asymmetry in the rates of electron and hole extraction^16–20^. Finally, heating of a plasmonic metal electrode surface, and conduction of heat through the surrounding medium, can affect electrochemical reactions. An elevated surface temperature can increase electron transfer kinetics and heating of the medium can increase diffusion coefficients and shift equilibrium reduction potentials. An underappreciated effect, detailed in this contribution, is the formation of a temperature gradient between the electrode surface and bulk electrolytic solution, which creates changes to the solution density, inducing convection^21–23^.

There are several reported methods to differentiate between thermal and nonthermal plasmonic contributions to electrochemical reactions. In most systems, an electrode is decorated with a large density of plasmonic particles. In most cases, the interparticle distance is too small for fast heat dissipation from spherical conduction to be assumed^24^. Willets and coworkers used scanning electrochemical microscopy to indirectly measure how large irradiation intensities can lead to shifts in equilibrium reduction potentials, accounting for a majority of the changes in their measured plasmon-enhanced electrochemical currents^25,26^. Hill and coworkers concluded from electrochemical simulations and chronoamperometry measurements that plasmonic photothermal heating enhances electrochemical currents by heat conduction and convection^24^. Commonly, chopped illumination of plasmonic electrodes is used to analyze the time-dependent responses of chronoamperograms^24,27^. In these experiments, fast current rise/fall times are attributed to hot charge carrier effects and slower current rise/fall times are attributed to photothermal heating effects. An issue with this approach is that rarely are chronoamperograms measured at different overpotentials to differentiate between electron transfer and mass transport effects. Additionally, there lacks sufficient evidence that the fast and slow current responses in plasmonic systems can solely be attributed to hot charge carriers and heating, respectively. Another common approach is to examine the functional form (e.g., linear, exponential) of the dependence of current density with light intensity^17,18,28^. However, the intensity range investigated is often small enough to obscure true functional relationships. Moreover, while different approaches are collectively increasing awareness of the prominence of plasmonic photothermal heating effects in electrochemistry^6,24–27,29–34^, and urging caution in misassigning electrochemical enhancement mechanisms, there is a lack of proper control experiments that isolate and allow quantification of photothermal heating. A common control that is practiced is to heat the electrolyte bath to an elevated temperature, which does not accurately produce the heating conditions in plasmonic electrochemistry experiments where the electrode surface is selectively heated, creating a temperature gradient that extends away from the surface into the bulk solvent^16,18,27^.

In the present study, we examine the role of photothermal heating by plasmonic excitation of nanoscale roughened Au electrodes in the simple outer-sphere electrochemical reduction and oxidation of Ru(NH_3_)_6_^3+/2+^. Cyclic voltammetry at different scan rates is used to investigate both electron transfer kinetics and mass transport-limiting current density. The response of plasmonically excited electrodes is compared with experiments that utilize electrodes whose surface is selectively heated in dark conditions to control for the thermal responses of plasmonically excited electrodes. Finite-element simulations of cyclic voltammograms at isothermal and heated electrodes are presented that highlight the contributions of photothermal heating in plasmon-assisted electrochemical reactions. The dependence of excitation wavelength is also systematically investigated to differentiate the effects of plasmon excitation and direct interband excitation of Au. We find that the primary contribution to enhanced electrochemical currents from light excitation is from a temperature gradient that forms between the electrode surface and bulk electrolytic solution that induces convection. Interband transitions are found to exclusively heat the electrode while intraband (plasmon) excitations lead to photothermal heating and nonthermal contributions to the enhanced current density. These studies support the growing realization that photothermal heating by plasmon excitation is a factor that must be accounted for when interpreting plasmon-assisted electrochemical reactions. Our approach of selectively heating the electrode surface also puts forth a simple and straightforward way to control for photothermal heating effects.

## Results

### Electrochemical system

**Fig. 1:**
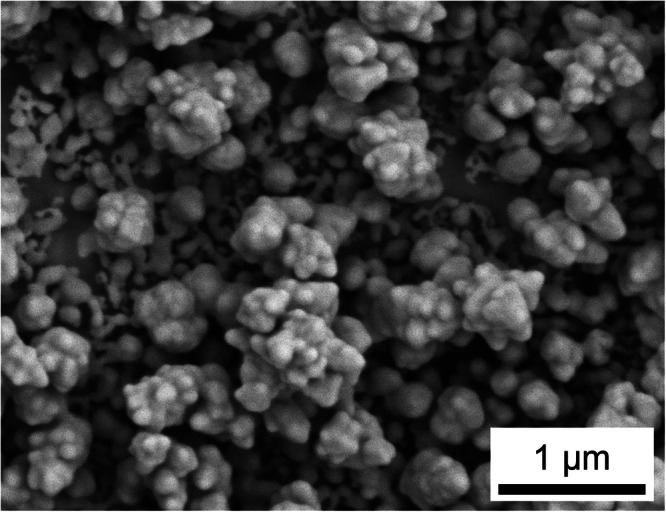
Image of plasmonic electrode. Representative scanning electron microscope image of an electrochemically roughened Au disk electrode.

To impart plasmonic activity onto a working electrode, we electrochemically roughened a mechanically polished Au disk electrode through a series of dissolution-redeposition cycles (see Methods for details). The redox cycling process results in a Au electrode with nanoscale roughness, appropriate for plasmon excitation with visible light (Figs. 1, S1, S2 and Table S1). Individual features on the electrode range from ca. 25 to 150 nm. Larger aggregates of nanoscale features span the length scale of ca. 100 to 500 nm. While the nanoscale features are not spatially uniform, the electrode surface is expected to have a broad plasmon resonance in the red to infrared portion of the electromagnetic spectrum due to the coupled plasmon resonances of individual, adjacent Au nanostructures^35,36^. To approximate the surface plasmon resonance of our electrodes, we prepared electrochemically roughened Au thin film substrates that mimic the surface of our electrochemically roughened Au disk electrodes. These substrates show ca. 25 to 150 nm individual nanoscale features that formed larger aggregates up to 500 nm, consistent with our electrode surfaces (Fig. S1). The absorbance spectra of the thin films (Fig. S3A) show a broad plasmon resonance at wavelengths greater than the interband threshold of ca. 520 nm for Au^37,38^. Due to the similarity in size of nanoscale features between the electrochemically roughened disk electrodes and thin films, we assume the plasmon resonances are similar, i.e., we assume a broad plasmon resonance spanning wavelengths greater than ca. 520 nm into the infrared for our electrochemically roughened Au electrodes.

The probe chosen for the current study is the Ru(NH_3_)_6_^3+/2+^ redox couple. This redox probe was chosen because it undergoes a facile and reversible one-electron, outer-sphere reaction to interconvert between the oxidation states of the metal. The majority of plasmon-assisted electrochemical reactions that have been reported are inner-sphere reactions that depend on adsorbate binding energies, surface chemistry, and newly formed electronic states from hybridized metal-adsorbate electronic states, all of which lead to a diversity of reported mechanisms of plasmon-assisted reactions^39–41^. An outer-sphere redox reaction is chosen here to simplify the mechanisms of plasmon-assisted electrochemistry to photothermal heating, irreversible charge transfer, photopotentials, and enhanced electromagnetic field effects. Plasmon-induced resonance energy transfer cannot occur in our system because the electronic absorption of the redox probe does not overlap with the plasmon resonance (Fig. S3). The Ru(NH_3_)_6_^3+/2+^ redox couple is in a buffered electrolytic solution in our system to eliminate potential competition from proton reduction.

### Effect of light excitation of a plasmonic electrode

**Fig. 2:**
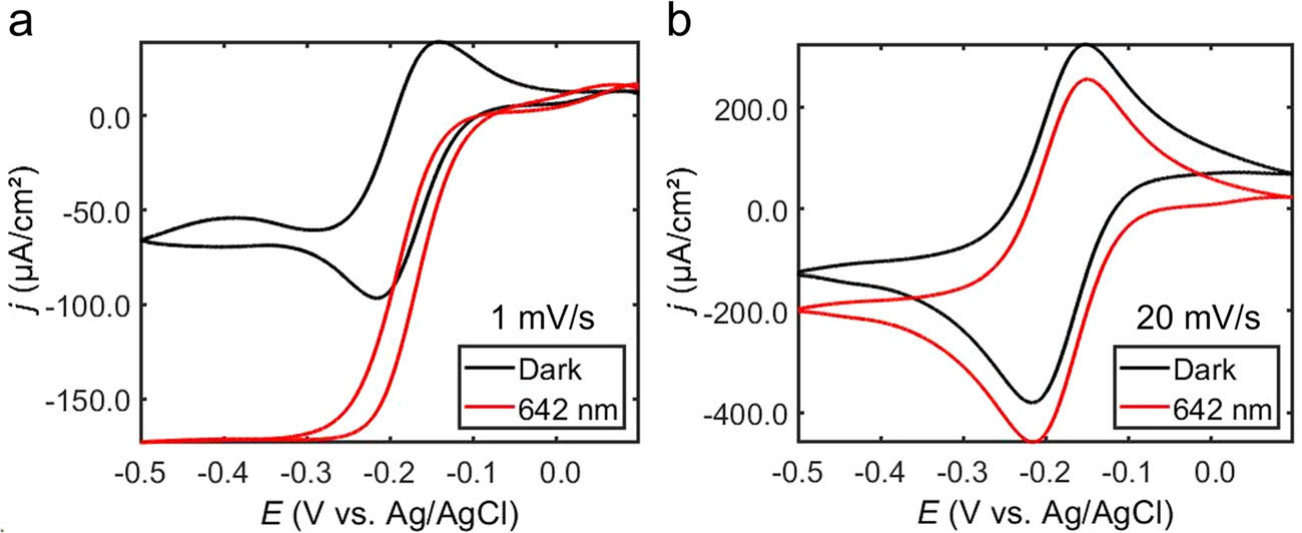
Cyclic voltammetry of Ru(NH_3_)_6_^3+/2+^ on plasmonic electrodes. Representative background-subtracted cyclic voltammograms measured at electrochemically roughened Au working electrodes in an electrolytic solution containing 0.1 M Na_2_HPO_4_·7H_2_O and 5 mM Ru(NH_3_)_6_Cl_3_·6H_2_O (pH = 6.0) with a scan rate of (**a**) 1 mV/s and (**b**) 20 mV/s. Black curves were obtained in dark conditions and red curves were obtained by irradiating Au electrodes with 2.45 W/cm^2^ of 642 nm laser light. A graphite rod was used as the counter electrode.

Cyclic voltammetry was used to probe the mechanisms of plasmon-assisted electrochemical reduction and oxidation of the Ru(NH_3_)_6_^3+/2+^ redox couple. To focus our study on the Faradaic reaction of the redox probe, all analyses were performed on background (electrolyte) subtracted cyclic voltammograms (unmodified, raw data is shown in the Supplementary Information). To excite the plasmon resonances of electrochemically roughened Au disk electrodes, without direct excitation of interband transitions in Au, 2.45 W/cm^2^ of circularly polarized 642 nm laser light was focused onto the geometric surface area of a Au disk. Cyclic voltammograms were then acquired at scan rates of 1, 5, and 20 mV/s. Figure 2 (Figs. S4–S6 and Table S2) compares the voltametric response of the Ru(NH_3_)_6_^3+/2+^ redox couple at electrochemically roughened Au disk electrodes in dark conditions with the electrode irradiated with light. In dark conditions, reversible peak-shaped voltammograms are measured as expected. Under plasmon excitation, the voltammograms acquired at 1 mV/s show a distinct reduction in the hysteresis of the anodic and cathodic waves (Fig. 2a) and an increase in current density in the mass transport-limiting regime of the curves. At 20 mV/s and under plasmon excitation conditions, the measured voltammograms show a similar hysteresis as those measured in dark conditions and smaller differences in current density (Fig. 2b). We note that the solution resistance does not appreciably change when the working electrode is in dark conditions or irradiated with light (Figs. S7–S9 and Tables S3–S9). In general, as the scan rate decreases, so too does the hysteresis in the voltammograms when the working electrode plasmon resonances are excited, along with an increase in current density (Figs. 2 and S5). This observation is consistent with previous reports of voltammograms acquired with heated electrodes^21,23,42–44^ (whereby a temperature gradient increases the concentration gradient resulting in an increase in the current density, a point that will be expanded on later), suggesting that photothermal heating is affecting the voltammogram shape in the present study.

Changes in the onset potential of an electrochemical reduction or oxidation reaction have been previously attributed to the establishment of a photopotential on the plasmonic electrode surface from light irradiation and to shifts in the equilibrium reduction potential of the redox probe due to photothermal heating^17,25^. Under light excitation, we measured less than a 10 mV change to the onset potentials for reduction and oxidation (Fig. S10). Onset potentials were determined using the third derivative of the current^16^. To determine if these potential shifts were due to an increase in temperature arising from plasmon excitation, we measured the electrode surface and bulk electrolytic solution temperature. To measure the temperature of the electrode surface, we inserted a thermocouple in the insulated interior of our working electrode (not exposed to the electrolytic solution) and in contact with the Au disk. (Fig. S11). In these temperature measurements, we assume that the Au disk temperature is constant across its 2.5 mm thickness. This assumption is validated by simulations of heat conduction across a 2.5 mm Au disk which show a temperature difference of <0.05 °C (Supplementary Methods 1, Fig. S12, and Table S10). To measure the bulk electrolyte temperature, we inserted a thermometer in the electrolytic solution ca. 10 mm away from the electrode surface and out of the path of the laser excitation beam. In dark conditions, the temperature of the electrochemically roughened Au disk electrode and the electrolytic solution was 23.0 °C (Table S11). When the electrode was irradiated with 2.45 W/cm^2^ of 642 nm laser light, the measured electrode surface temperature rose to 24.2 °C, while the electrolytic solution temperature remained constant within our detection limit (Table S11). The equilibrium reduction potential of Ru(NH_3_)_6_^3+/2+^ has been reported to shift 0.46 mV/K^45^. Based on a change in the electrode surface temperature of 1.2 K measured here, photothermal heating would shift the equilibrium potential <1 mV. Therefore, a temperature-induced change in the reduction potential is ruled out as the primary reason for the change in the onset potential. Rather, the change in the onset potentials, small in magnitude, may either be attributed to the establishment of a photopotential on the Au surface or attributed to errors in the determination of the onset potential. If a photopotential is established on Au, it likely arises from either the accumulation of hot charge carriers on the plasmonic electrode due to asymmetric electron and hole extraction rates or due to the plasmoelectric effect, where excitation off of peak plasmon resonance induces charge transfer to or from the metal, causing a change in its electron density and the buildup of a photopotential. Optical rectification is unlikely due to the relatively modest illumination intensity used in this study^9^. Due to the small magnitude of the change in onset potential, we conclude that if a photopotential is developed on Au, it contributes minimally to the observed changes in the electrochemistry. Additionally, excitation of the plasmon resonances with light enhanced the heterogeneous electron transfer rate constant by ca. 30% relative to the rate constant measured with the electrode surface set at 24.2 °C (Fig. S13 and Table S12).

**Fig. 3:**
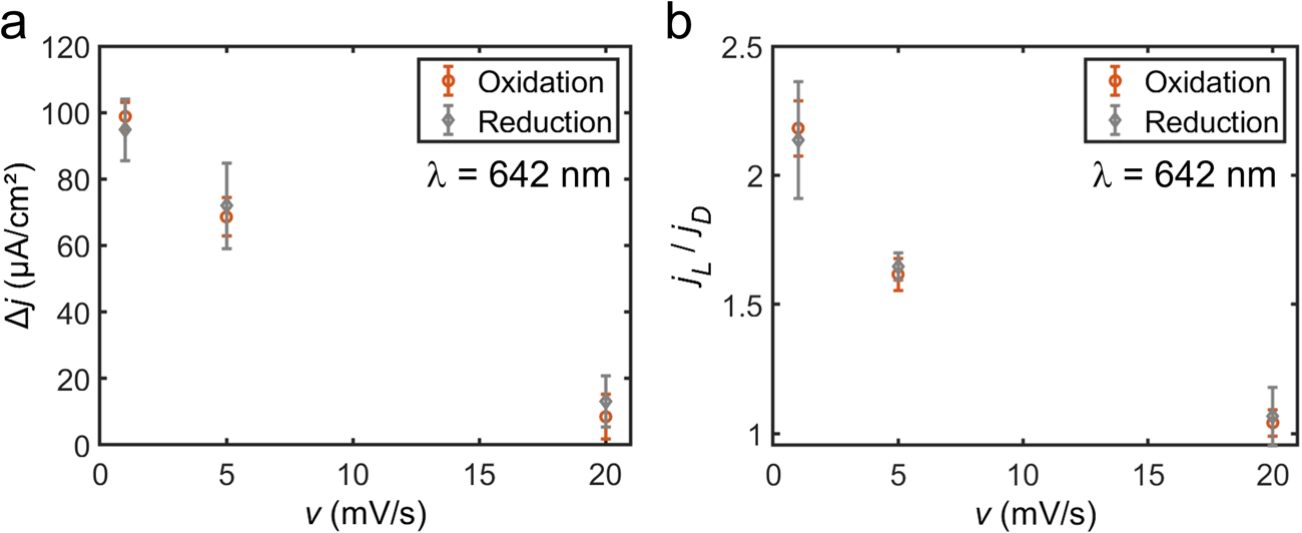
Light-enhanced current density. **a** Change in the background subtracted current density (Δj) and (**b**) current density (j) enhancement for the oxidation (orange data) and reduction (gray data) of the Ru(NH_3_)_6_^3+/2+^ redox probe as a function of scan rate when electrochemically roughened Au electrodes were irradiated with 2.45 W/cm^2^ of 642 nm laser light (j_L_) and in dark conditions (j_D_). Δj *=* j_L_ – j_D_. Data points are average values from three independent trials and the errors bars represent the standard deviations of the measurements.

A second metric of cyclic voltammograms that is useful in parsing the mechanisms of plasmon-assisted electrochemistry is the current density in the mass transport-limiting regime. Oxidation and reduction current densities in the mass transport-limiting regime were tabulated at applied potentials of 0.05 and −0.45 V vs. Ag/AgCl (3 M KCl), respectively (Fig. S14). We measure a near-linear increase in the difference in current density (Δ*j*) measured under light irradiation (*j*_L_) and dark conditions (*j*_D_) and corresponding exponential increase in the current density enhancement (*j*_L_ / *j*_D_) under light irradiation with a decrease in scan rate (Fig. 3). The light-enhanced current density is also similar between the oxidation and reduction reactions of the redox probe, indicating that the redox reactions share an enhancement mechanism. Enhancement in the mass transport-limiting regime may arise from increases in the diffusion coefficients, induced convection, enhanced migration of the electroactive species, or a combination thereof. Thus, although charge carriers may shift the onset potential, light-enhanced current density in this regime, along with the change in hysteresis of the anodic and cathodic waves, suggests that photothermal heating is a primary contributor in the plasmon-assisted electrochemistry of the system under study.

**Fig. 4:**
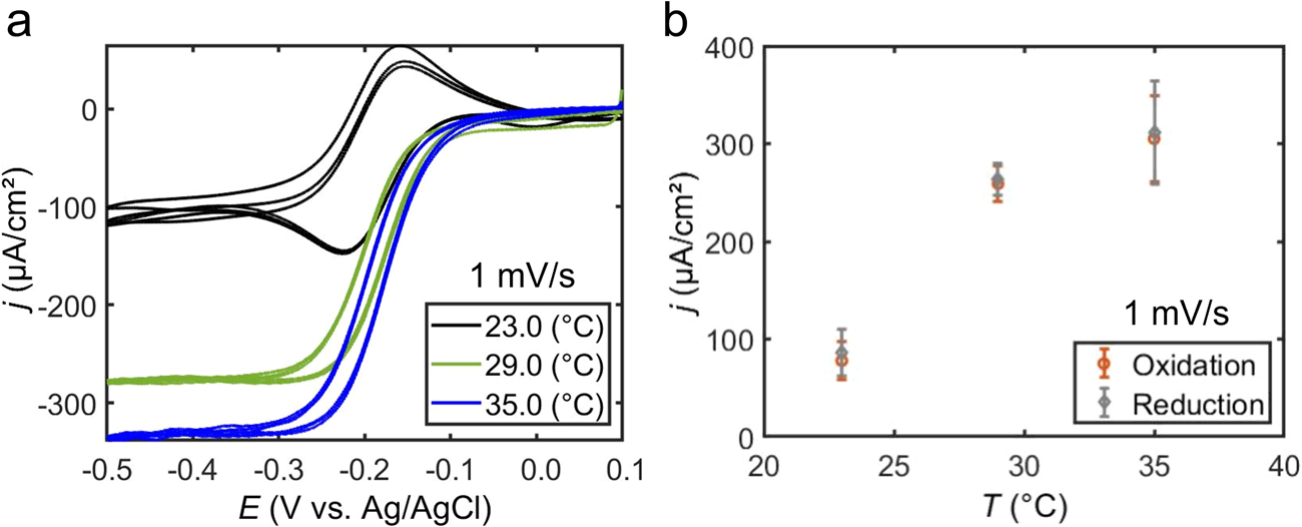
Heated electrode voltammetry. **a** Representative background subtracted cyclic voltammograms measured at electrochemically roughened Au working electrodes in an electrolytic solution containing 0.1 M Na_2_HPO_4_·7H_2_O and 5 mM Ru(NH_3_)_6_Cl_3_·6H_2_O (pH = 6.0) in dark conditions as a function of set electrode surface temperature. **b** Mass transport-limiting current density (j) measured as a function of set electrode surface temperature. Scan rate is 1 mV/s. A graphite rod was used as the counter electrode.

To support the assertion that photothermal heating of the electrode is a primary contributor to plasmon-assisted electrochemistry, we employed a nanoscale roughened Au disk working electrode where the Au surface temperature could be controlled and measured (Fig. S11). We collected cyclic voltammograms with a scan rate of 1 mV/s at electrode surface temperatures ranging from 23.0 °C to 35.0 °C (Figs. 4a, S15, and S16) in dark conditions. From these voltammograms, we observe that an increase in electrode surface temperature results in an increase in the mass transport-limiting current density (Fig. 4b) and a decrease in the hysteresis of the anodic and cathodic waves. That is, the presence of a temperature gradient between the electrode surface and bulk electrolytic solution changes the shape of the voltammogram, and the magnitude of the gradient determines the mass transport-limiting current density. We note that this control experiment closely mimics a plasmonic electrode irradiated with light, where heating is localized to the electrode surface and dissipated through the system. A common and less accurate control in plasmon-assisted electrochemistry is to set the bulk electrolytic solution temperature (Fig. S17), which precludes the development of a temperature gradient. Another common experiment used to separate hot charge carrier from photothermal heating effects is to measure chronoamperometry under chopped illumination. Similar to previous reports, chronoamperograms acquired under chopped illumination of our system show slow current rise and fall times, a characteristic usually attributed to photothermal effects (Fig. S18). However, as detailed below, our control experiments using a heated electrode reveal that photothermal heating is not the sole mechanism responsible for the increase in current density over these long time scales. Previous reports employing heated electrodes have posited that the presence of a temperature gradient induces convection and reduces the hysteresis in the cathodic and anodic waves of voltammograms acquired from kinetically facile redox reactions^21,23,42–44^. From similar trends observed in the voltammetry data acquired from a plasmonically excited electrode and from an electrode surface heated in dark conditions, we conclude that photothermal heating plays a significant role in plasmon-assisted electrochemistry. This fact is often overlooked, or improperly controlled for, in favor of attributing electrochemical enhancement primarily to plasmonically generated hot charge carriers.

**Fig. 5:**
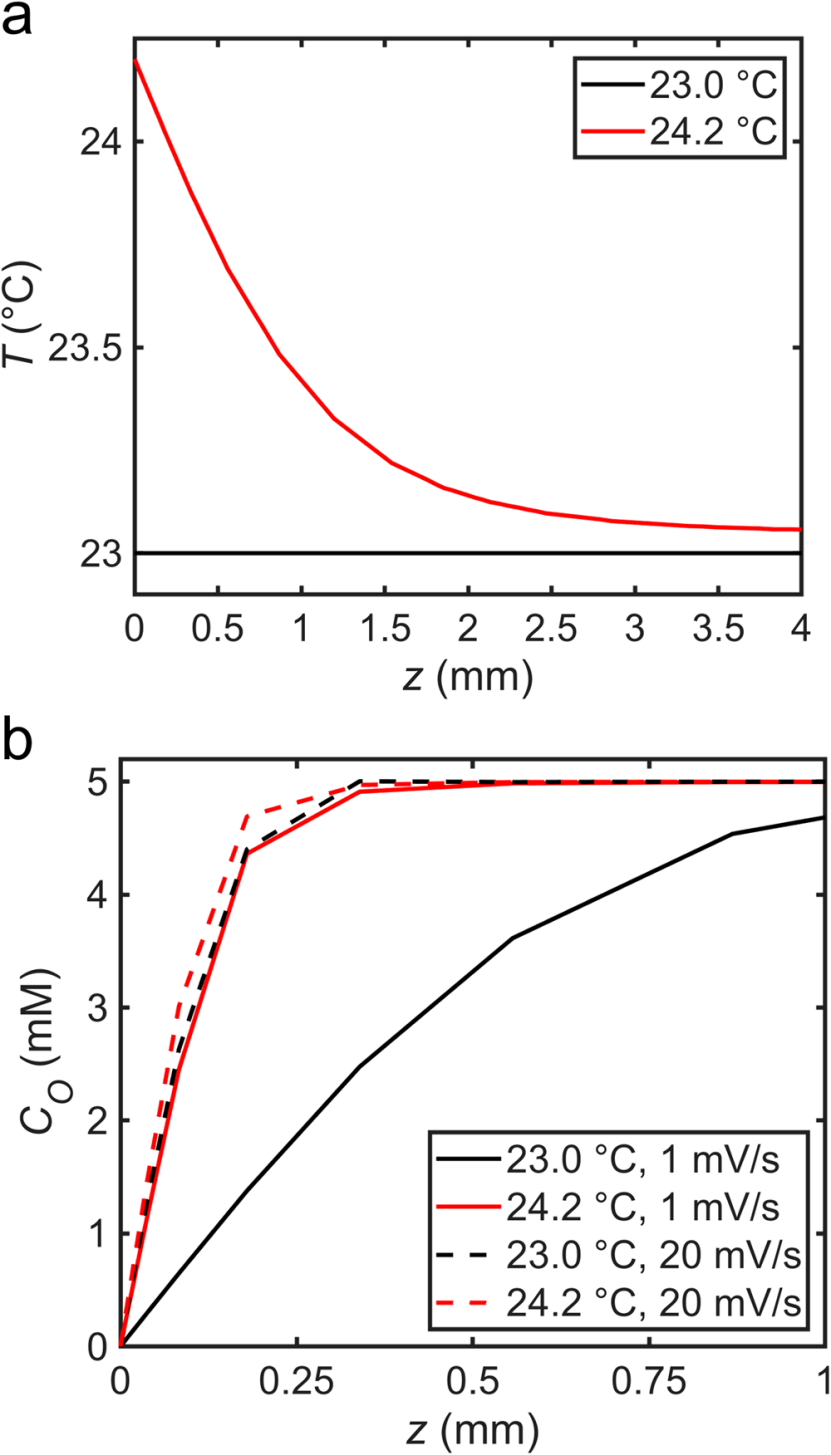
Temperature and concentration gradients at a heated electrode. One-dimensional (**a**) temperature and (**b**) concentration gradients obtained from 2D-axisymmetric finite-element simulations of Ru(NH_3_)_6_^3+^ electrochemical reduction at steady-state conditions with the electrode surface temperature set at 23.0 °C (black curves) and 24.2 °C (red curves). The aqueous solvent temperature is set to an initial temperature of 23.0 °C. Concentration gradients in panel B are shown for scan rates of 1 mV/s (solid lines) and 20 mV/s (dashed lines). *z* indicates distance away from the origin of the electrode surface (*z* *=* 0 mm).

A temperature gradient between the electrode surface and bulk electrolytic solution is formed when plasmonic electrodes are irradiated with light (Table S11). This gradient is present and constant across all cyclic voltammetry measurements from plasmonic electrodes irradiated with a fixed wavelength and intensity of light. However, as the scan rate increases, the steady-state diffusion layer decreases. Although the diffusion coefficients of the redox probe increase when the electrode is irradiated with light compared to in dark conditions due to photothermal heating (Figs. S4, S19–S22), and may vary across the temperature gradient, the diffusion coefficients of the redox probe are unchanged when comparing data collected when the wavelength and intensity of excitation light are held constant (e.g., Fig. 2). Therefore, a comparison of the temperature gradient with the concentration gradient at different scan rates can provide insight into how photothermal heating enhances mass transport-limiting current. Figure 5 (also see Figs. S23–S25) shows finite-element simulations (Supplementary Methods 2) of the temperature and concentration gradients formed at steady-state conditions when the electrode surface temperature is equal to or 1.2 °C greater than the bulk electrolytic solution temperature and when the electrode potential is set to a reducing potential of −0.45 V vs. Ag/AgCl. Because heat transfer is faster than mass transfer, the temperature gradient extends further from the electrode surface than the concentration gradient. At a scan rate of 20 mV/s, the diffusion layer, and the temperature gradient across this layer, is relatively small. The relatively small temperature gradient across the diffusion layer results in only a small reduction of the diffusion layer. At 1 mV/s, the diffusion layer grows to a length comparable to the temperature gradient. In this case, the temperature gradient causes substantial mixing within the diffusion layer (convection), effectively reducing its extension away from the electrode surface, and enhances the measured current density in the mass transport-limiting regime. This convection is driven by differences in the solution density caused by the temperature gradient present in the electrolytic solution. Near the electrode surface, where the temperature is greatest, the solution density is lower than in the bulk electrolytic solution. Higher density electrolytic solution is driven to the electrode surface as the lower density electrolytic solution rises to electrolyte–air interface.

**Fig. 6:**
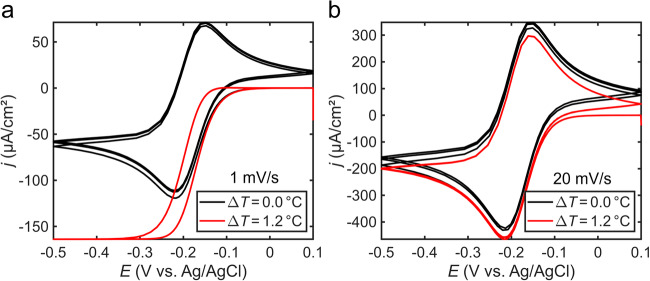
Simulated cyclic voltammograms. Cyclic voltammograms simulated in an aqueous solvent containing a reversible redox couple with characteristics of Ru(NH_3_)_6_^3+/2+^ at a scan rate of (**a**) 1 mV/s and (**b**) 20 mV/s. Black curves were obtained with the electrode surface and solution temperature set at 23.0 °C (ΔT *=* 0 °C). Red curves were obtained with the electrode surface temperature set at 24.2 °C and the solution temperature set at 23.0 °C (ΔT *=* 1.2 °C). Three potential cycles were simulated for each condition.

To further validate that the presence of a temperature gradient is responsible for the change in shape and current density in our cyclic voltammetry measurements, we simulated cyclic voltammograms obtained from an electrode with its surface heated to the temperature we measured under 2.45 W/cm^2^ of 642 nm light irradiation (Supplementary Methods 2). First, with the electrode surface and bulk electrolytic solution at the same temperature of 23.0 °C, simulated cyclic voltammograms obtained at scan rates of 1 and 20 mV/s (Fig. 6, black curves) match well with experimentally obtained cyclic voltammograms of our redox probe (Fig. 2). Introducing a temperature gradient in the simulation, by setting the electrode surface 1.2 °C greater than the bulk electrolyte temperature, also produced voltammograms (Fig. 6, red curves) that matched well with the experimentally measured cyclic voltammograms obtained when the working electrode is irradiated with 642 nm light. In particular, the reduction in hysteresis between the anodic and cathodic waves at 1 mV/s was obtained in the simulation of the heated electrode as well as the increasing difference in mass transport-limiting current density between a heated electrode (irradiated with light) and an electrode isothermal with the bath with decreasing scan rate. Simulated cyclic voltammograms produced at a scan rate of 1 mV/s with the electrode surface and bath at an equal and elevated temperature (24.2 or 30.2 °C) do not yield voltammograms with a reduction in hysteresis or increase in current density (Fig. S26) because the diffusion coefficients do not significantly change over this temperature range (Fig. S22A), highlighting the impact of a temperature gradient in cyclic voltammetry measurements. The strong agreement between the simulation and experiment, along with the control experiment using heated electrodes, provides evidence for photothermal heating as a primary contributor to the plasmon-enhanced electrochemistry in our system. Remarkably, these results indicate that a temperature gradient as small as ca. 1 °C induces strong enough convection to significantly alter the voltametric response. Much larger temperature gradients, and enhanced current densities, are expected to contribute to the enhancement of other reported plasmon-enhanced electrochemical reactions, which often use intensities much larger than the relatively modest intensities used in the present study.

### Effect of excitation light wavelength in plasmon-assisted electrochemistry

**Fig. 7:**
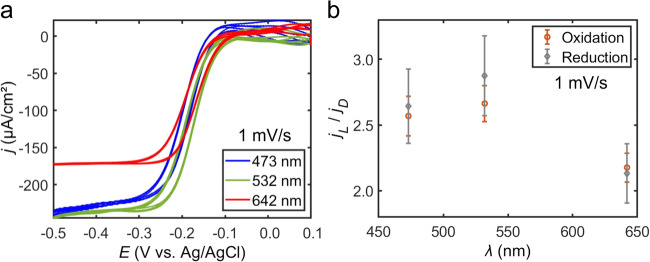
Wavelength dependent cyclic voltammetry. **a** Representative background subtracted cyclic voltammograms measured at electrochemically roughened Au working electrodes in an electrolytic solution containing 0.1 M Na_2_HPO_4_·7H_2_O and 5 mM Ru(NH_3_)_6_Cl_3_·6H_2_O (pH = 6.0) when irradiated with 2.45 W/cm^2^ of 473 nm laser light (blue curves), 532 nm laser light (green curves), or 642 nm laser light (red curves). **b** Enhancement in the current density for the oxidation (orange data) and reduction (gray data) of the Ru(NH_3_)_6_^3+/2+^ redox probe as a function of excitation wavelength measured at electrochemically roughened Au electrodes. Data points are average values from three independent trials and the errors bars represent the standard deviations of the measurements. Scan rate is 1 mV/s. A graphite rod was used as the counter electrode.

Light excitation of electrochemically roughened Au electrodes can excite plasmon resonances or directly excite interband transitions within Au, depending on the light excitation energy. Direct excitation of interband transitions results in deexcitation pathways that parallel plasmon decay, namely the formation of energetic electron-hole pairs followed by electron and phonon scattering events that release heat to the surrounding medium. To compare the impact of plasmon-assisted electrochemistry and Au photoelectrochemistry, we measured cyclic voltammograms of the Ru(NH_3_)_6_^3+/2+^ redox probe at excitation wavelengths of 473, 532, and 642 nm (Figs. 7, S5, S6, and S27–S32). The threshold for interband transitions in Au is ca. 520 nm^37,38^. Therefore, light irradiation of 473 nm excites interband transitions, and light irradiation of 532 or 642 nm excites the Au plasmon resonance of the electrochemically roughened electrode. Interband excitation of Au (473 nm) results in an enhanced mass transport-limiting current density and a decrease in the anodic and cathodic wave hysteresis as is observed with plasmon excitation (Figs. 7a and S31). In comparison with plasmon excitation at 642 nm, 473 nm light excitation produces larger absolute current densities (Figs. 7a and S33) as well as enhanced current densities (Figs. 7b and S34). Excitation at 532 nm results in current densities greater than excitation at 642 nm and similar current densities compared to excitation at 473 nm. No statistical difference was measured in the light-induced change of the reduction and oxidation onset potentials of the Ru(NH_3_)_6_^3+/2+^ redox probe as a function of excitation wavelength (Figs. S34 and S35). These observations indicate that photothermal heating through interband transitions also has a substantial effect on the redox reaction.

**Fig. 8:**
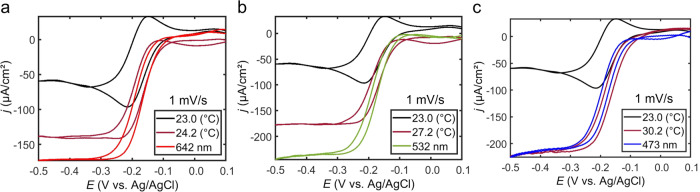
Photothermal controls for plasmon-assisted voltammetry. Background subtracted cyclic voltammograms measured at electrochemically roughened Au working electrodes in an electrolytic solution containing 0.1 M Na_2_HPO_4_·7H_2_O and 5 mM Ru(NH_3_)_6_Cl_3_·6H_2_O (pH = 6.0) at a scan rate of 1 mV/s. Black curves were obtained in dark conditions at room temperature (23.0 °C) and maroon curves were obtained in dark conditions while heating the electrode surface to a temperature equivalent to the surface temperature measured under light irradiation with 2.45 W/cm^2^ of (**a**) 642 nm laser light, (**b**) 532 nm laser light, and (**c**) 473 nm laser light. Red, green, and blue curves were obtained by irradiating the electrodes with 2.45 W/cm^2^ of 642, 532, and 473 nm laser light, respectively. Black curves in A-C are the same data shown for comparison. A graphite rod was used as the counter electrode.

**Fig. 9:**
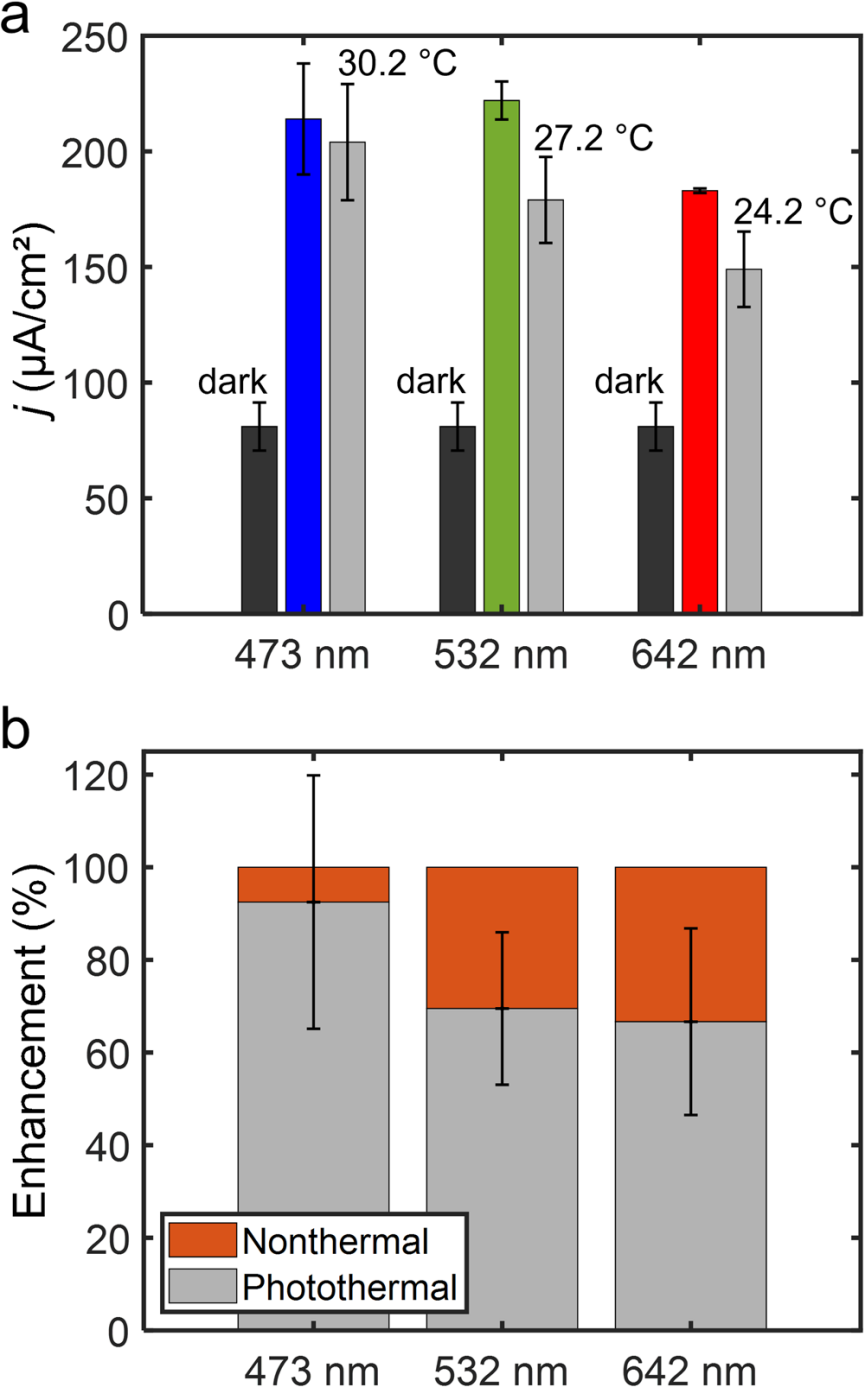
Quantification of plasmon-enhanced voltammetry. **a** Ru(NH_3_)_6_^2+^ oxidation current densities tabulated at 0.05 V vs. Ag/AgCl from background subtracted cyclic voltammograms acquired at electrochemically roughened Au disk electrodes. Dark gray bars are data collected in dark conditions (reproduced for each wavelength for comparison), colored bars are data collected when irradiating plasmonic working electrodes with 2.45 W/cm^2^ of 473 nm (blue), 532 nm (green), or 642 nm (red) laser light. Light gray bars are data collected in dark conditions with the working electrode temperature set at 30.2 °C, 27.2 °C, or 24.2 °C, corresponding to the working electrode temperature measured when irradiated with the respective wavelengths of light. Data points are average values from three independent trials and the errors bars represent the standard deviations of the measurements. **b** Contributions of nonthermal effects (orange) and photothermal heating (light gray) to the enhanced oxidation current densities when the plasmonic electrodes are irradiated with light.

Next, we quantified the contributions of photothermal heating to the light-enhanced electrochemistry for direct interband (473 nm excitation) transitions and plasmon excitation (532 and 642 nm excitation) with cyclic voltammetry (Figs. 8 and S36). Using our electrode with a measurable and controllable surface temperature, we measured the electrode surface temperature when electrochemically roughened Au electrodes in an electrolytic solution were irradiated with 2.45 W/cm^2^ of 473, 532, or 643 nm light (Table S11). The electrode surface temperature increases monotonically with a decrease in excitation wavelength, while the bulk electrolyte temperature remained constant for all wavelengths (Table S11). Simulations of cyclic voltammograms at heated electrodes with temperature gradients corresponding to temperatures measured at electrodes irradiated with light are in generally good qualitative agreement with our experimental data (Figs. 8 and S37–S39). The one exception is that simulations predict that an electrode heated by excitation light at 473 nm should yield the highest current density, but our experimental data show 473 and 532 nm light excitation produce similar current densities (Figs. 9a and S40). A comparison of data from voltammograms acquired at 1 mV/s in dark conditions at room temperature with voltammograms acquired under light irradiation and with voltammograms acquired in dark conditions at an electrode surface temperature set to the temperature measured under light excitation is shown in Fig. 9a (Fig. S40). With these measurements, the heated electrode in dark conditions controls for the photothermal contribution to the current density enhancement when the electrodes are irradiated with light. With all excitation wavelengths, photothermal heating is a primary contributor to the enhanced current density. In the case where only interband transitions occur, photothermal heating accounts for nearly all of the enhanced current density. When plasmon resonances of the electrode are excited, photothermal heating accounts for a majority (ca. 65–70%), but not all, of the enhancement (Figs. 9b and S40). Because these analyses are made using data from the mass transport-limiting regime, the balance of current density enhancement is not attributed to electrostatic potentials on the electrode produced from light excitation or direct participation of hot charge carriers in the redox events. The reason for the additional current density enhancement under plasmon excitation conditions not due to photothermal heating is not known at this time and is currently under further investigation. Potential reasons may include enhanced migration or enhanced transport due to optical forces^46–49^ due to the large electromagnetic field gradients at the plasmonic electrode surface.

## Discussion

Excitation of a plasmonic Au electrode with light generates hot electron-hole pairs. These energetic charge carriers are either produced via direct excitation of Au via interband transitions or through electronic transitions caused by the decay of plasmon resonances. Small changes to the onset potential under light excitation of the electrode suggest that if a photopotential is established on the plasmonic Au electrode, it does not appreciably affect the electrochemistry of the electroactive species, Ru(NH_3_)_6_^3+/2+^, in the present study. The remaining hot charge carriers relax through scattering, resulting in the heating of the electrode surface and eventual transfer of heat to the surrounding medium. Under continuous illumination, the electrode surface reaches an elevated, steady-state temperature while the surrounding solvent quickly dissipates the heat resulting in a negligible rise in its temperature. The formed temperature gradient between the electrode surface and bulk solvent induces free convection, decreasing the diffusion layer thickness, and resulting in an increase in the steady-state current density from the reduction or oxidation reaction. As the wavelength of light decreases, the energy of the hot charge carriers increases, resulting in larger temperature gradients and larger enhancements in current density. It should be noted that although plasmon excitation concentrates light, the majority of stored energy is released radiatively by light scattering.

The standard heterogeneous electron transfer rate constant of the redox probe increases when the plasmonic electrode resonance is excited with light; an elevated electrode surface temperature does not account for the rate constant increase under plasmon excitation (Fig. S13). The change in temperature from light excitation of a plasmonic electrode marginally increases the diffusion coefficients (Fig. S22A), which does not account for the enhancement in current density measured. Photothermal heating is responsible for the enhanced current density observed from direct interband transitions. Photothermal heating caused by excitation of plasmon resonances accounts for >50% of the enhanced current density. The balance of the current density enhancement is not fully understood but may be a result of optical forces or enhanced migration due to the large electromagnetic field gradients concentrated at the plasmonic electrode surface further enhancing the transport of the redox species to the electrode surface.

In summary, we have detailed the effects of exciting Au plasmonic electrodes with visible light using the outer-sphere Ru(NH_3_)_6_^3+/2+^ redox couple as a probe. Proper control experiments, aided by simulations, demonstrate that photothermal heating is a primary contributor to the enhancement in plasmon-assisted electrochemistry. A key fundamental advance in this study is that the formation of a temperature gradient in plasmon-assisted electrochemistry can significantly enhance the reaction rate due to induced convection. A temperature gradient as low as ca. 1 °C can enhance the current density measured by >2×. Heating of Au was found to be the exclusive enhancement mechanism when light directly promoted interband transitions. Excitation of the plasmon resonances results in hot charge carriers, electrostatic potentials, heating, and enhanced local electromagnetic fields. The latter two are the primary contributors of mass transport-limiting current density enhancements when the excitation light frequency overlaps with the plasmon resonance of the electrode. In addition to fundamental insight, this study provides a method to control for and study the contributions of photothermal heating in plasmon-assisted electrochemistry.

## Methods

### Materials

Hexaamineruthenium (iii) chloride (Ru(NH_3_)_6_Cl_3_·6H_2_O, 98.0%, lot #A0408729) was purchased from Acros Organics. Sodium phosphate dibasic heptahydrate (Na_2_HPO_4_·7H_2_O, 98%, lot #0995C456) was purchased from VWR Life Science. Alumina powders (0.3 µm, lot #210727 and 0.05 µm, lot #210628) were purchased from Electron Microscopy Sciences. Type 1 water was obtained from an ultrapure water system (Sartorius, Arium mini). Nitric acid (HNO_3_, lot #MKCM2152) and potassium chloride (KCl, lot #SLBP3785V) were purchased from Sigma-Aldrich. Au disk electrodes (Ø = 2 mm) and Ag/AgCl (3 M KCl) reference electrodes were purchased from CH Instruments. A universal 1/32 DIN PID temperature controller, 12 V AC/DC (part #SYL-1612B) was purchased from Auber Instruments. A VITEK power supply: 12 V DC, 100/240 V AC (part #54DL52), and silicone heat sink compound (part #44N787) were purchased from Grainger. An aluminum bar (12 mm × 12 mm, part #7083T42) was purchased from McMaster-Carr. A cartridge heater (12 V, 40 W ceramic cartridge, part #M-GVJ-EE2H) was purchased from MatterHackers. Type K thermocouple probes (Ø = 1.6 mm, part #61161-372) and glass coverslips (24 × 60 mm, No. 1, part #48404-455) were purchased from VWR International. Gold pellets (Au, 99.999% pure, lot #1001306) were purchased from Kurt J. Lesker. Platinum wire (Pt, part #10956-BS) was purchased from Alfa Aesar.

### Preparation of mechanically polished electrodes

A Au disk electrode was first polished using aqueous slurries of 0.3 μm alumina particles on a cloth pad, and then sonicated in ultrapure H_2_O. Next, the Au electrode was polished using aqueous slurries of 0.05  μm alumina particles on a cloth pad, followed by sonication in ultrapure H_2_O, and then dried with N_2_ gas.

### Preparation of electrochemically roughened electrodes

A mechanically polished Au electrode was first electrochemically cleaned by cycling the applied potential between −0.25 V and 1.5 V vs. Ag/AgCl (3 M KCl) for 25 cycles at 100 mV/s in 0.1 M H_2_SO_4_. A Pt mesh was used as the counter electrode. Next, the Au electrode was electrochemically roughened by an oxidative dissolution‒reductive deposition cycle using a procedure based on previous work^50^. In a solution of 0.1 M KCl, the Au working electrode potential was modulated between −0.3 V (held for 3 s) and +1.2 V (held for 1.2 s) for 75 cycles. A Pt mesh and Ag/AgCl (3 M KCl) electrode were used as the counter and reference electrodes, respectively.

### Preparation and optical characterization of thin film electrodes

Au thin films were fabricated by depositing a thin (ca. 150 nm) Au film directly onto no. 1 glass coverslips at a rate of 1 Å/s in an AXXIS electron beam thin film deposition system (Kurt J. Lesker). Roughened Au thin films were prepared using a roughening procedure based on previous work^50^: a three-electrode electrochemical cell setup was employed, in which the Au thin film functioned as the working electrode and was partially submerged in 0.1 M KCl. Pt and Ag/AgCl (3 M KCl) served as counter and reference electrodes, respectively. Using a potentiostat in cyclic-step chronoamperometry mode, the applied potential was stepped to −0.3 V for 3 s and +1.2 V for 1.2 s for 90 cycles. Electronic absorbance spectra were acquired using a Cary 60 UV-Vis spectrophotometer (Agilent Technologies). The sample was positioned perpendicular to the incident beam, which was scanned from 800 to 200 nm, at 600 nm/min.

### Scanning electron microscopy characterization of electrodes

Images were obtained using an Apreo 2C field emission scanning electron microscope (Thermo Fischer Scientific) using an Everhard-Thornley detector (ETD). An accelerating voltage of 5.00 kV and beam current of 25 pA were used. Thin film samples were imaged in secondary electron mode. Copper tape was used to allow current to flow from the thin films to the stage. The thin film samples were tilted 35° relative to the normal of the primary electron beam. Elemental analysis and imaging were conducted using a QUANTAX Energy-Dispersive X-ray (EDX) detector (Bruker). A voltage of 30 kV and a spot size of 10 were used. Images were collected 8.5 mm normal from the electrode surface.

### Electrolyte preparation

20 mL of 0.1 M Na_2_HPO_4_·7H_2_O and 5 mM Ru(NH_3_)_6_Cl_3_·6H_2_O was used as the electrolytic solution, adjusted to a pH value of 6.0 using HNO_3_.

### Electrochemical measurements

A single compartment, three-electrode system with a Au working electrode, a graphite rod counter electrode, Ag/AgCl (3 M KCl) reference electrode, and an electrolytic solution containing 0.1 M Na_2_HPO_4_·7H_2_O and 5 mM Ru(NH_3_)_6_Cl_3_·6H_2_O (pH = 6.0) was used for cyclic voltammetry measurements conducted with a 660E CH Instruments potentiostat. Prior to electrochemical measurements, each working electrode was stabilized by cycling the potential between 0.1 and −0.5 V at 100 mV/s for 25 cycles under light irradiation (the wavelength used for stabilization corresponds to the wavelength under study), followed by cycling the potential for 25 additional cycles in the same potential window in dark conditions. For light irradiation, 2.45 W/cm^2^ of 642 nm, 532 nm, or 473 nm circularly polarized laser light was focused onto the working electrode, ensuring the laser spot size matched the geometric surface area of the working electrode. Data was collected in order of increasing scan rate. For each scan rate (1 mV/s, 5 mV/s, and 20 mV/s), measurements were made first under light irradiation followed by in dark conditions. Control experiments in dark conditions were performed using an electrically heated Au electrode (Fig. S11). All measurements were carried out in triplicate on separately prepared working electrodes. Chronoamperometry measurements were made at a fixed potential of –0.45 V. The illumination was chopped (i.e., modulated between on and off) by manually shuttering the laser at 30 s intervals.

### Electrochemical impedance spectroscopy

Impedance measurements were made using a 660E CH Instruments potentiostat at two dc voltages (–0.45 V and 0.05 V). All measurements used an ac voltage of 10 mV, and the frequency ranged from 1000 kHz to 50 kHz. In order to extract the solution resistance, charge transfer resistance, and double-layer capacitance, simple equivalent circuits were built using the CH Instruments software to fit the raw electrochemical impedance data.

### Data analysis

Cyclic voltammograms were analyzed using an in-house algorithm written in MATLAB. Onset potentials were determined using the third derivative of the current^16^. Oxidation and reduction currents were determined by tabulating baseline-corrected current values at +0.05 V and –0.45 V, respectively. The percentage of photothermal and nonthermal contributions to the enhanced current density were determined by a ratio of current densities obtained by heating an electrode in dark conditions to current densities obtained by irradiating an electrode with light, each less the current density obtained in dark conditions at 23.0 °C.

### Simulations

Finite-element simulations of the electrochemical system were performed using COMSOL Multiphysics, version 6.1. Full details of the model and simulations can be found in the Supplementary Information.

### Supplementary information

Supplementary Information

## Data Availability

The authors declare that all the data supporting the findings of this study are available within the article (and Supplementary Information Files), or available from the corresponding author on reasonable request.

## Code availability

Data processing code and simulation models are available from the corresponding author on reasonable request.
