# Supplementary material for: Quantification and description of photothermal heating effects in plasmon-assisted electrochemistry
Source: Commun Chem. 2024 Apr 1;7:70. doi: 10.1038/s42004-024-01157-8 (PMC10984925; doi:10.1038/s42004-024-01157-8)
Supplement: Supplementary file 1 — Supplementary Information [file 42004_2024_1157_MOESM1_ESM.pdf]

SUPPLEMENTARY INFORMATION

Quantification and description of photothermal heating effects in plasmon-assisted electrochemistry

Md. Al-Amin,[1] Johann V. Hemmer,[1] Padmanabh B. Joshi,[1,2] Kimber Fogelman,[1] and Andrew J. Wilson[1]*

[1]Department of Chemistry, University of Louisville, Louisville, Kentucky, 40292, United States

[2]Present address: Duke University, Durham, NC, 27708, United States

*Corresponding author e-mail: aj.wilson@louisville.edu

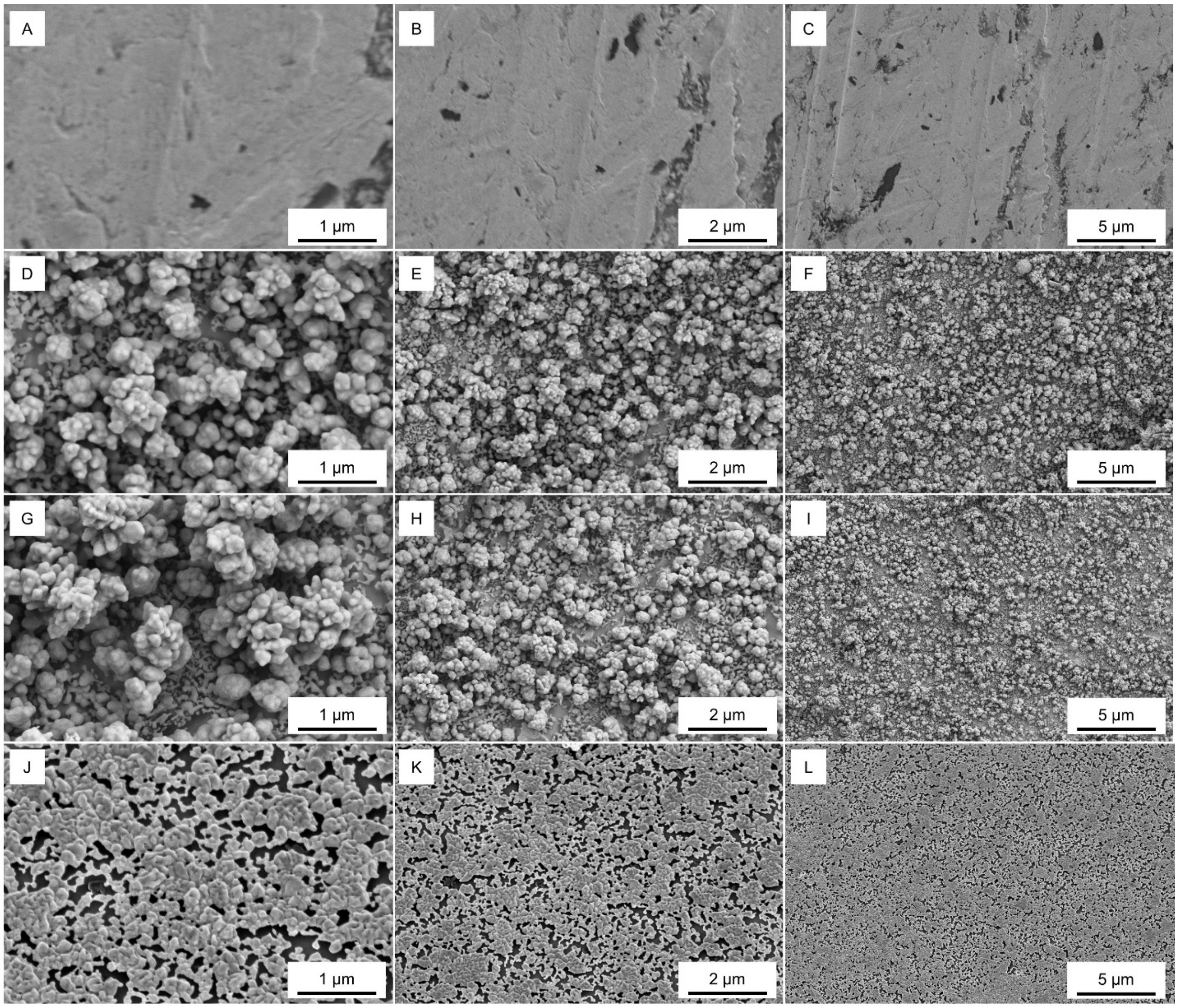

Figure S1. Scanning electron microscopy images at different magnifications of (A-C) a mechanically polished Au electrode, (D-F) an electrochemically roughened Au electrode before light irradiation, (G-I) an electrochemically roughened Au electrode after cycling the potential of working electrode from 0.1 V to -0.5 V vs. Ag/AgCl (3 M KCl) in an electrolytic solution containing 0.1 M $Na_2HPO_4 \cdot 7H_2O$ and 5 mM $Ru(NH_3)_6Cl_3 \cdot 6H_2O$ (pH = 6.0) at a scan rate of 1, 5, and 20 mV/s and continuous electrode irradiation with 2.45 W/cm^2 of 532 nm laser light, and (J-L) an electrochemically roughened thin film Au electrode.

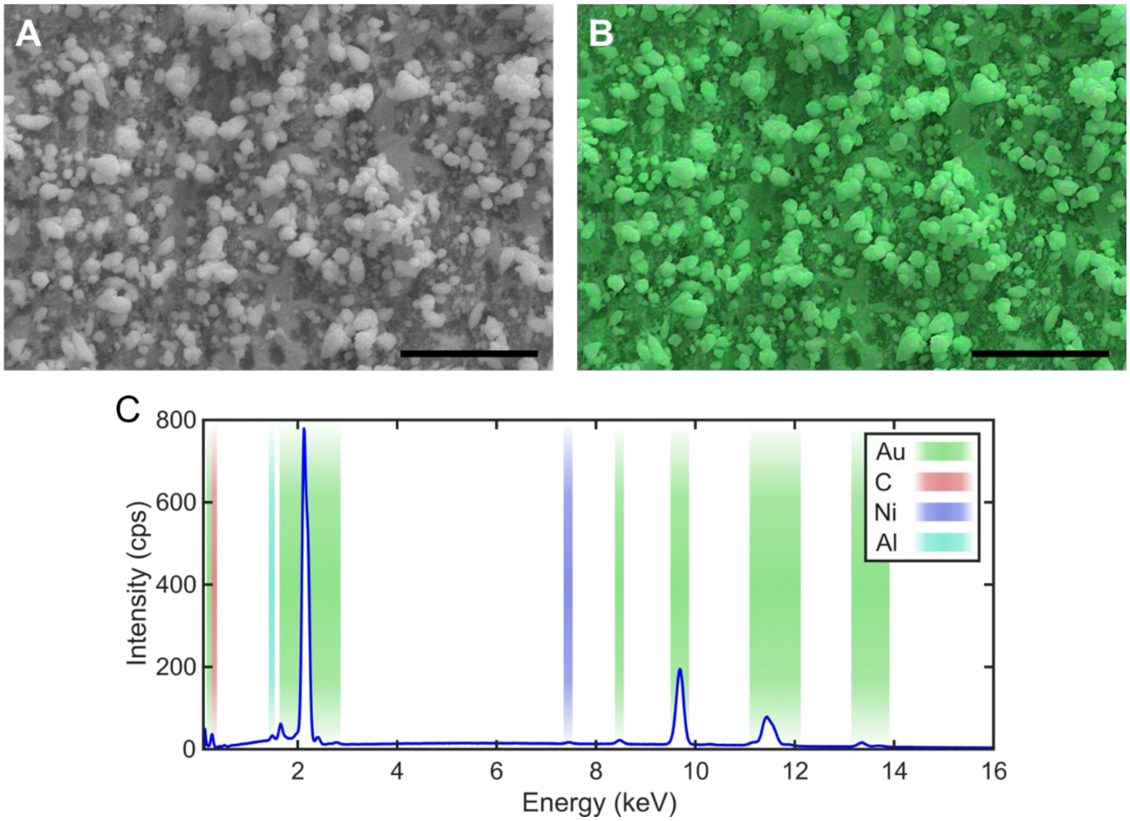

Figure S2. (A) Scanning electron microscopy image of an electrochemically roughened Au disk electrode. Energy-dispersive X-ray (B) map and (C) spectrum acquired from the electrode region shown in panel A. The map shows an absolute scale based on the relative abundance of the elements color coded and listed in the legend of panel C. Scale bars are 5 μm.

Table S1. Mass percentages of major elements measured by energy-dispersive X-ray spectroscopy on an electrochemically roughened Au disk electrode.

Element	Area	Mass (%)
Au	907612	83.86
C	25549	15.53
Al	9358	0.36
Ni	6940	0.25

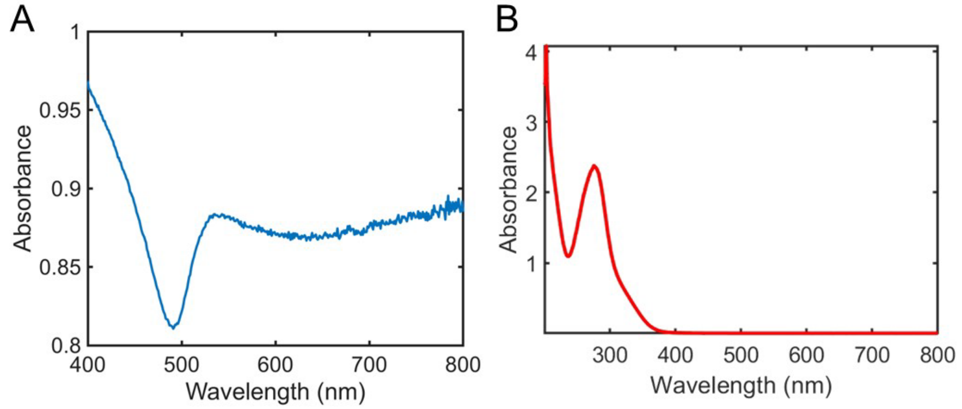

Figure S3. Absorbance spectra of (A) an electrochemically roughened Au thin film and (B) 0.1 M $Na_2HPO_4 \cdot 7H_2O$ and 5 mM $Ru(NH_3)_6Cl_3 \cdot 6H_2O$ (pH = 6.0).

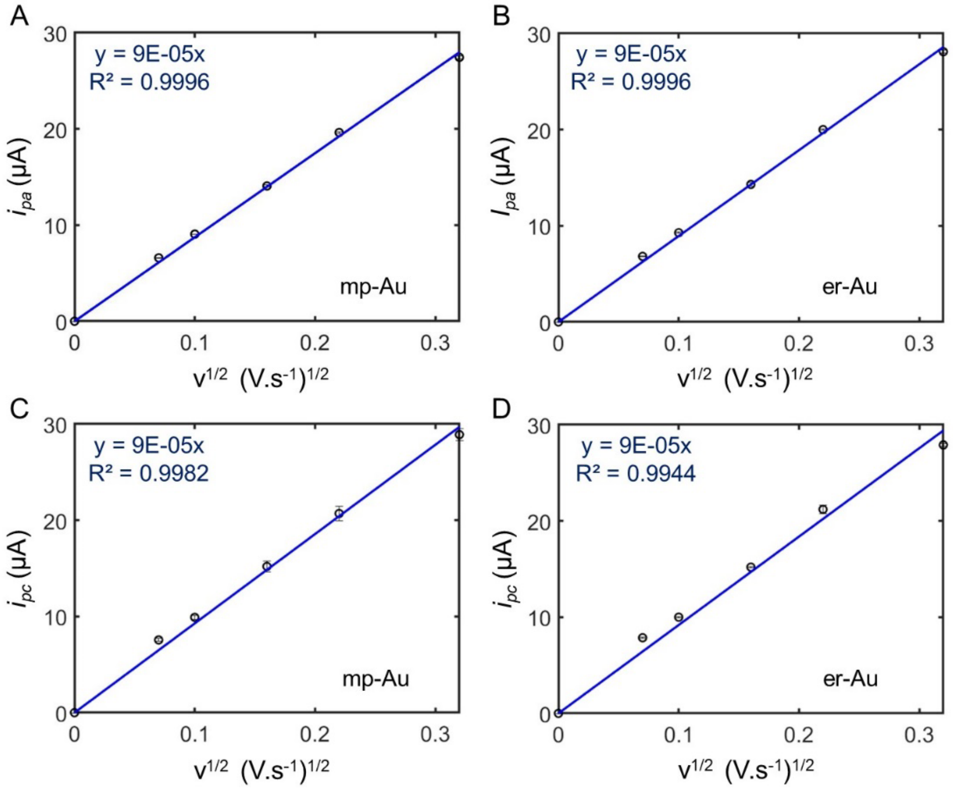

Figure S4. (A, B) Anodic and (C, D) cathodic peak currents (i_{pa} and i_{pc}, respectively) obtained from cyclic voltammograms measured at (A,C) mechanically polished Au (mp-Au) and (B, D) electrochemically roughened Au (er-Au) working electrodes in dark conditions in an electrolytic solution containing 0.1 M $Na_2HPO_4 \cdot 7H_2O$ and 5 mM $Ru(NH_3)_6Cl_3 \cdot 6H_2O$ (pH = 6.0) as a function of the square root of the scan rate (v). Data points are measured values, and the solid lines represent linear fits to the data.

Supplementary Note 1.

The Randles-Ševčík equation and the geometric surface area of a mechanically polished Au electrode (0.031 cm^2) was used to determine the diffusion coefficients of the reduced and oxidized form of $Ru(NH_3)_6^{3+/2+}$. The diffusion coefficients were then used to determine the electrode area active for the redox probe of the electrochemically roughened Au electrodes.

Table S2. Diffusion coefficients (D) of the $Ru(NH_3)_6^{3+/2+}$ redox couple determined from the Randles-Ševčík plots and the geometric surface area of mechanically polished Au working electrodes. Electrode surface area (SA) of electrochemically roughened (er) Au working electrodes determined by the Randles-Ševčík plot and the diffusion coefficients of $Ru(NH_3)_6^{3+/2+}$.

	**D (cm^2/s)**	**SA er-Au (cm^2)**
**Anodic peak current**	$4.49 \pm 0.3 \times 10^{-6}$	0.0321 ± 0.002
**Cathodic peak current**	$5.31 \pm 0.6 \times 10^{-6}$	0.0317 ± 0.001
**Average**		0.0319 ± 0.001

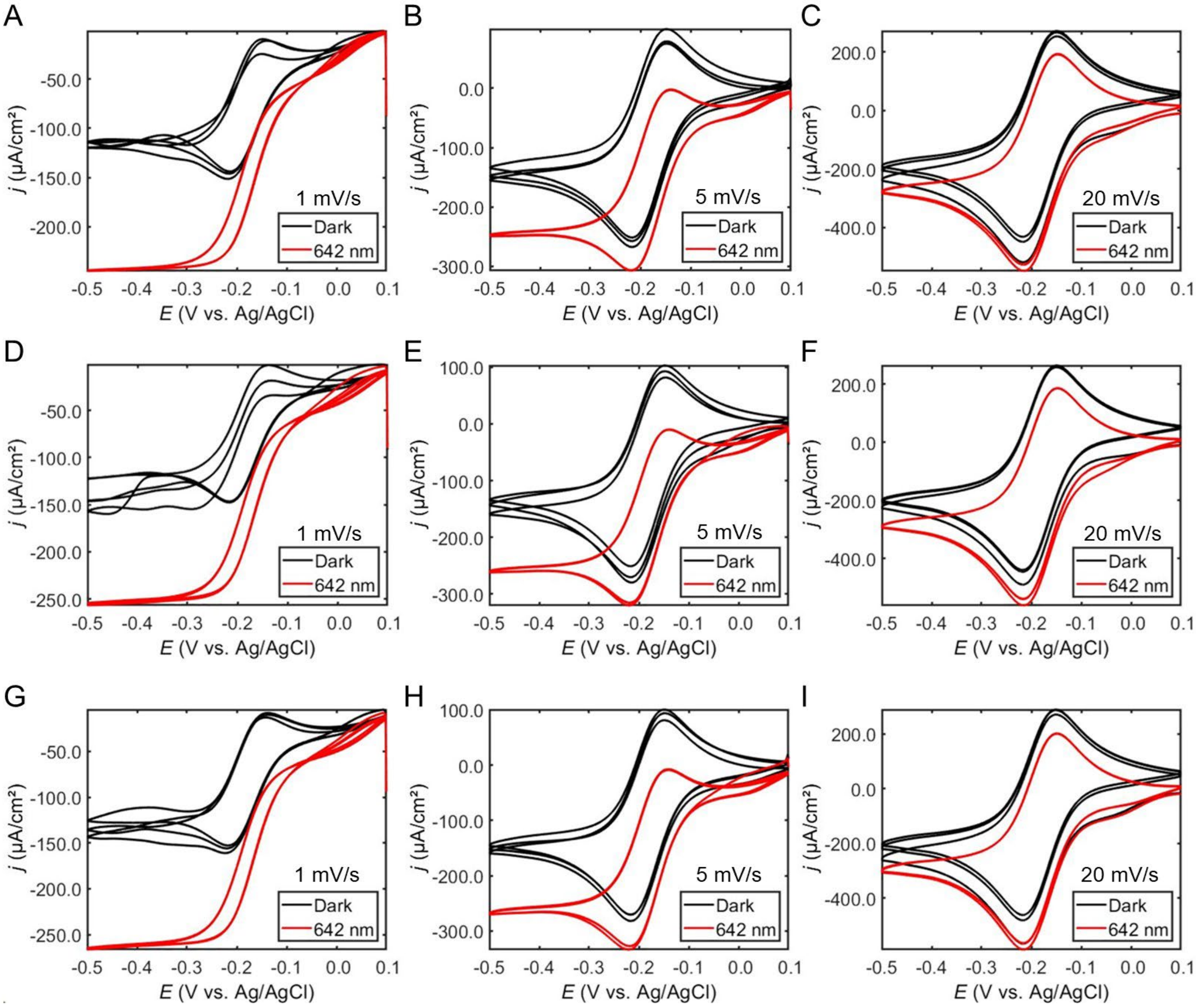

Figure S5. Cyclic voltammograms measured at electrochemically roughened Au working electrodes in an electrolytic solution containing 0.1 M $Na_2HPO_4 \cdot 7H_2O$ and 5 mM $Ru(NH_3)_6Cl_3 \cdot 6H_2O$ (pH = 6.0) with a scan rate of (A) 1 mV/s, (B) 5 mV/s, and (C) 20 mV/s. (D-F) and (G-I) Repeat trials of panels A-C on separately prepared Au electrodes. Black curves were obtained in dark conditions and red curves were obtained by irradiating Au electrodes with 2.45 W/cm^2 of 642 nm laser light. A graphite rod was used as the counter electrode.

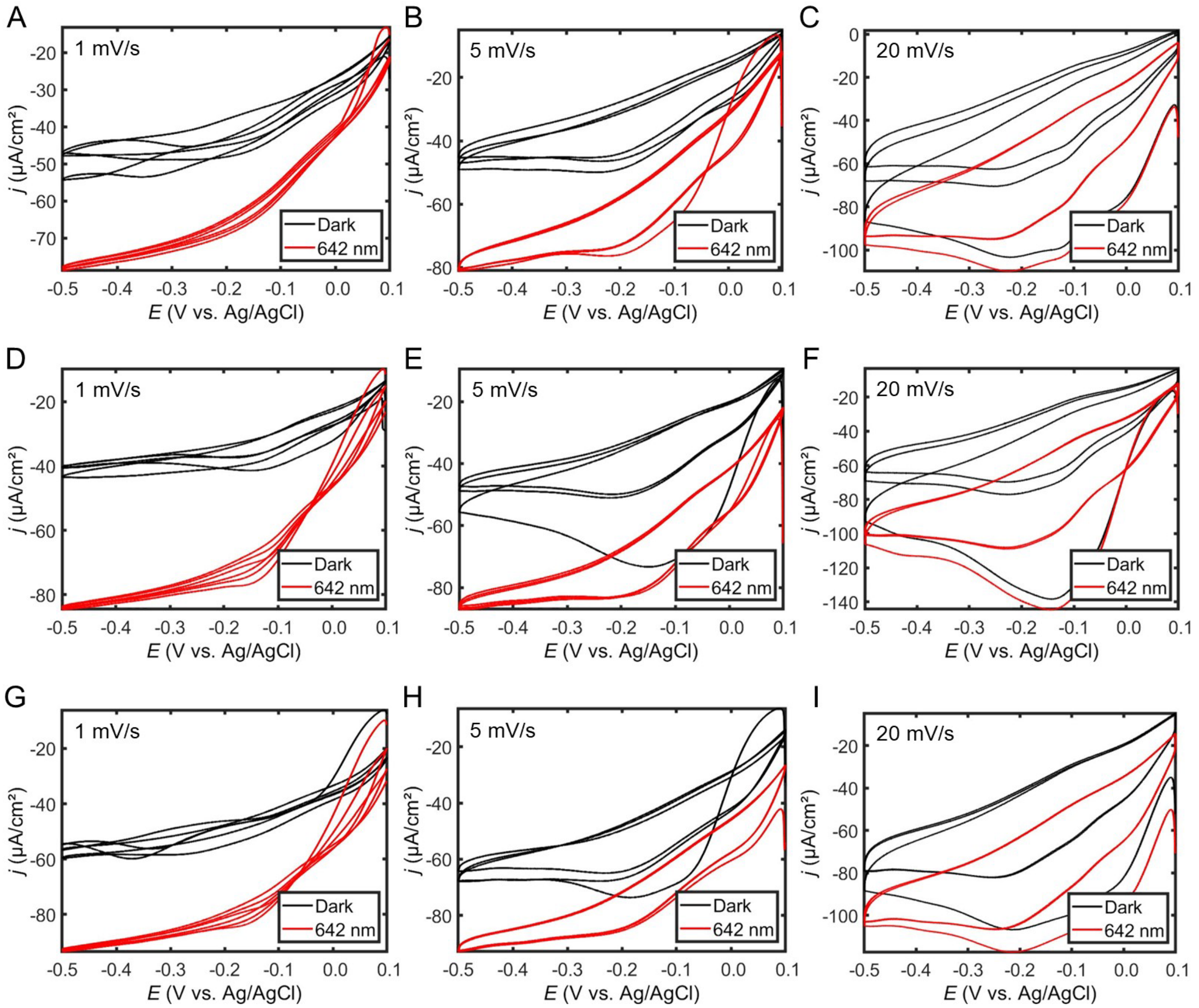

Figure S6. Cyclic voltammograms measured at electrochemically roughened Au working electrodes in an electrolytic solution containing 0.1 M $Na_2HPO_4 \cdot 7H_2O$ (pH = 6.0) with a scan rate of (A) 1 mV/s, (B) 5 mV/s, and (C) 20 mV/s. (D-F) and (G-I) Repeat trials of panels A-C on separately prepared Au electrodes. Black curves were obtained in dark conditions and red curves were obtained by irradiating Au electrodes with 2.45 W/cm^2 of 642 nm laser light. A graphite rod was used as the counter electrode.

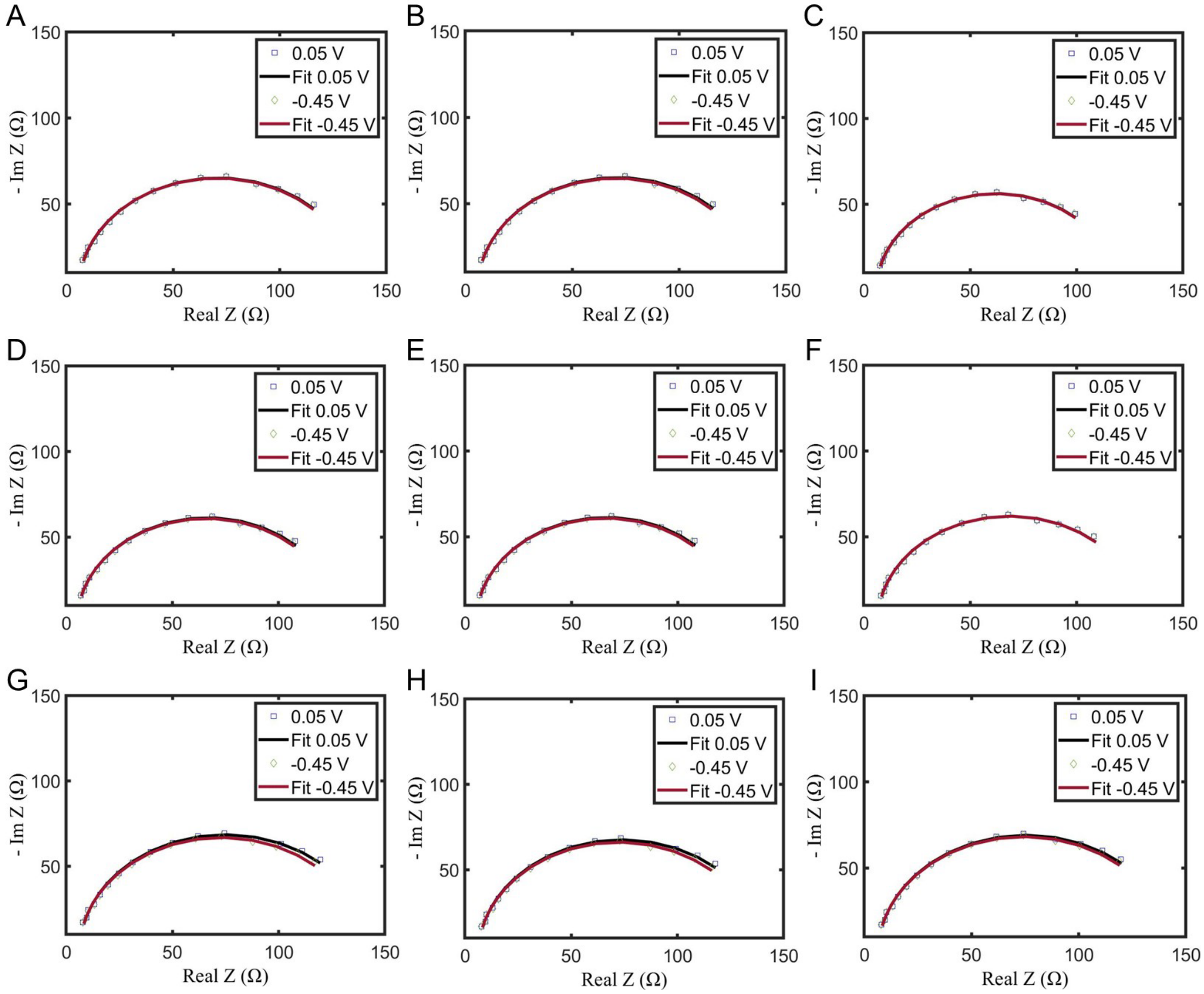

Figure S7. Complex-plane plots from EIS measurements using mechanically polished Au disk electrodes acquired with a dc voltage of -0.45 V and 0.05 V, and with an ac voltage of 10 mV. Panels A–C highlight three trials for mechanically polished Au electrodes under 2.45 W/cm^2 of 642 nm laser irradiation. Panels D–F highlight three trials for mechanically polished electrodes under 2.45 W/cm^2 of 532 nm laser irradiation. Panels G–I highlight three trials for mechanically polished Au electrodes under 2.45 W/cm^2 of 473 nm laser irradiation. EIS measurements were obtained in a solution containing 0.1 M $Na_2HPO_4 \cdot 7H_2O$ and 5 mM $Ru(NH_3)_6Cl_3 \cdot 6H_2O$ (pH = 6.0) with a mechanically polished Au electrode under light irradiation conditions. Square and diamond symbols are raw data and solid lines are fits to a Randles equivalent circuit. A graphite rod was used as the counter electrode. Ag/AgCl (3 M KCl) was used as the reference electrode.

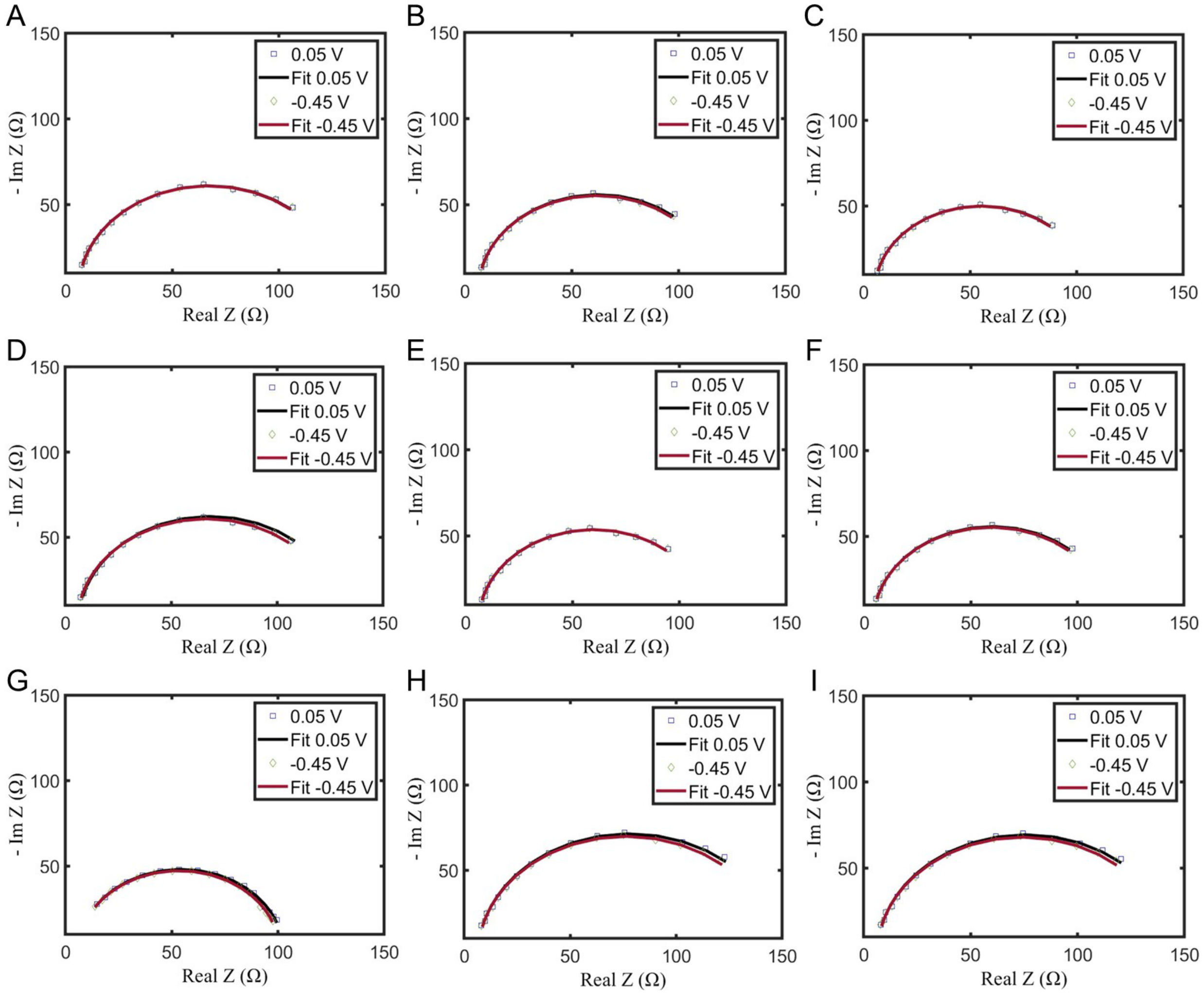

Figure S8. Complex-plane plots from EIS measurements using electrochemically roughened Au disk electrodes acquired with a dc voltage of -0.45 V and 0.05 V, and with an ac voltage of 10 mV. Panels A–C highlight three trials for electrochemically roughened Au electrodes under 2.45 W/cm^2 of 642 nm laser irradiation. Panels D–F highlight three trials for electrochemically roughened Au electrodes under 2.45 W/cm^2 of 532 nm laser irradiation. Panels G–I highlight three trials for electrochemically roughened Au electrodes under 2.45 W/cm^2 of 473 nm laser irradiation. EIS measurements were obtained in a solution containing 0.1 M $Na_2HPO_4 \cdot 7H_2O$ and 5 mM $Ru(NH_3)_6Cl_3 \cdot 6H_2O$ (pH = 6.0) with electrochemically roughened Au electrodes under light irradiation conditions. Square and diamond symbols are raw data and solid lines are fits to a Randles equivalent circuit. A graphite rod was used as the counter electrode. Ag/AgCl (3 M KCl) was used as the reference electrode.

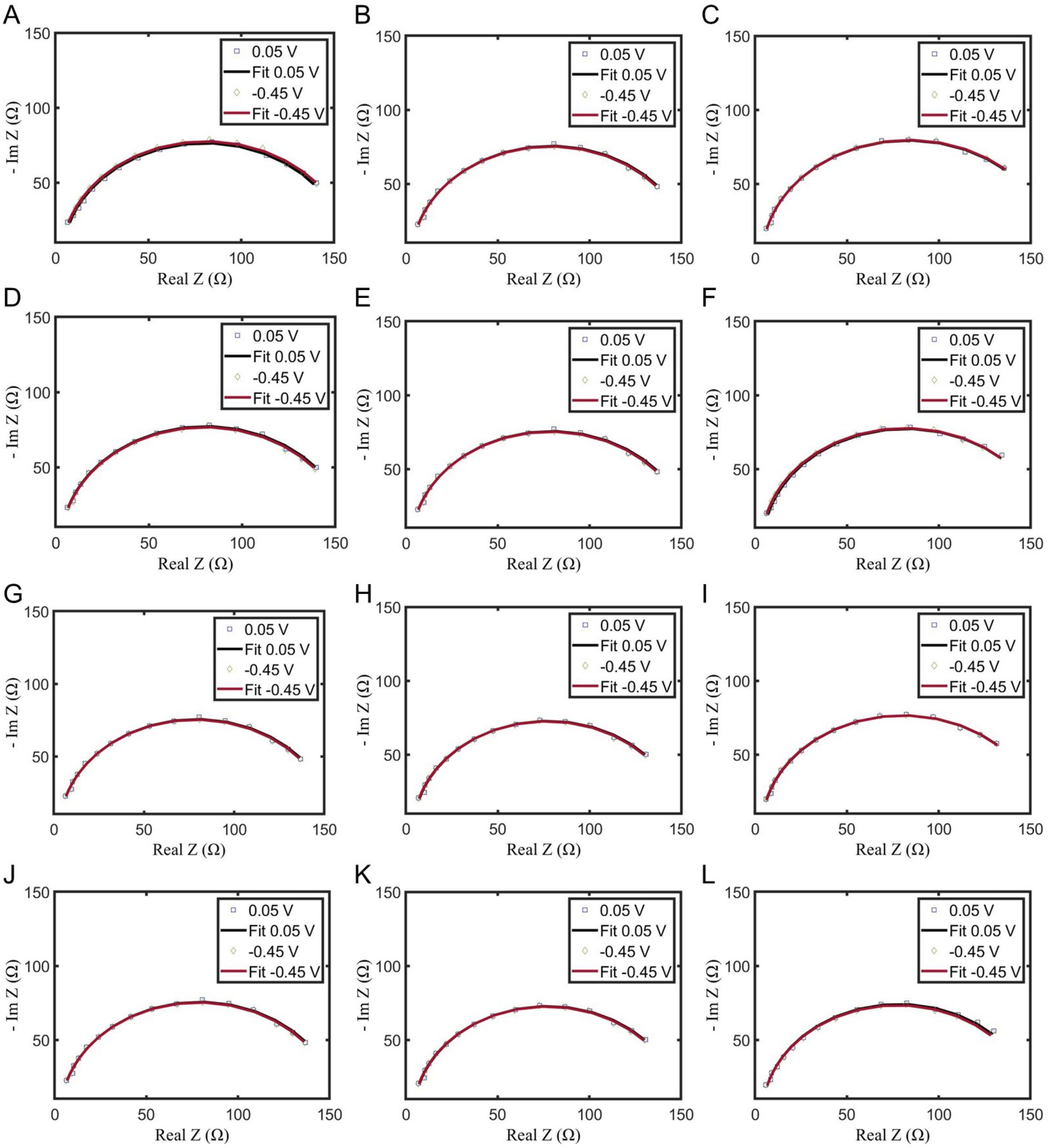

Figure S9. Complex-plane plots from EIS measurements using electrochemically roughened Au disk electrodes in dark conditions with a dc voltage of –0.45 V and 0.05 V, and with an ac voltage of 10 mV. Panels A–C highlight three trials for an electrode surface temperature set at 23.0 °C. Panels D–F highlight three trials for an electrode surface temperature set at 24.2 °C. Panels G–I highlight three trials for an electrode surface temperature set at 27.2 °C. Panels J–K highlight three trials for an electrode surface temperature set at 30.2 °C. EIS measurements were obtained in a solution containing 0.1 M $Na_2HPO_4 \cdot 7H_2O$ and 5 mM $Ru(NH_3)_6Cl_3 \cdot 6H_2O$ (pH = 6.0). Square and diamond symbols are raw data and solid lines are fits to a Randles equivalent circuit. A graphite rod was used as the counter electrode. Ag/AgCl (3 M KCl) was used as the reference electrode.

Table S3. Solution resistance (R_S), charge transfer resistance (R_{CT}), and double layer capacitance (C_{dl}) determined by fitting electrochemical impedance spectroscopy data to a simple Randles equivalent circuit. Measurements were made in an electrolytic solution containing 0.1 M $Na_2HPO_4 \cdot 7H_2O$ and 5 mM $Ru(NH_3)_6Cl_3 \cdot 6H_2O$ (pH = 6.0) at oxidizing (0.05 V vs. Ag/AgCl) and reducing (-0.45 V vs. Ag/AgCl) dc potentials with an ac potential of 10 mV on a mechanically polished Au working electrode that was irradiated with 2.45 W/cm^2 of 642 nm laser light. A graphite rod was used as the counter electrode.

Trial	R_S (Ω)		R_{CT} (Ω)		C_{dl} (nF)	
	0.05 V	-0.45 V	0.05 V	-0.45 V	0.05 V	-0.45 V
1	5.7	5.6	130.4	130.3	9.2	9.2
2	5.7	5.6	130.4	129.5	9.3	9.3
3	6.0	5.9	112.5	112.4	11.3	11.3
Avg.	5.8	5.7	124.4	124.1	9.9	9.9
Std. dev.	0.1	0.1	8.4	8.3	1.0	1.0

Table S4. Solution resistance (R_S), charge transfer resistance (R_{CT}), and double layer capacitance (C_{dl}) determined by fitting electrochemical impedance spectroscopy data to a simple Randles equivalent circuit. Measurements were made in an electrolytic solution containing 0.1 M $Na_2HPO_4 \cdot 7H_2O$ and 5 mM $Ru(NH_3)_6Cl_3 \cdot 6H_2O$ (pH = 6.0) at oxidizing (0.05 V vs. Ag/AgCl) and reducing (-0.45 V vs. Ag/AgCl) dc potentials with an ac potential of 10 mV on an electrochemically roughened Au working electrode that was irradiated with 2.45 W/cm^2 of 642 nm laser light. A graphite rod was used as the counter electrode.

Trial	R_S (Ω)		R_{CT} (Ω)		C_{dl} (nF)	
	0.05 V	-0.45 V	0.05 V	-0.45 V	0.05 V	-0.45 V
1	6.1	6.0	122.0	121.9	11.0	10.8
2	6.3	6.2	111.8	110.6	12.0	12.4
3	5.2	5.2	100.1	99.3	13.1	11.6
Avg.	5.9	5.8	111.3	110.6	12.0	11.6
Std. dev.	0.5	0.4	8.9	9.2	0.9	0.7

Table S5. Solution resistance (R_S), charge transfer resistance (R_{CT}), and double layer capacitance (C_{dl}) determined by fitting electrochemical impedance spectroscopy data to a simple Randles equivalent circuit. Measurements were made in an electrolytic solution containing 0.1 M $Na_2HPO_4 \cdot 7H_2O$ and 5 mM $Ru(NH_3)_6Cl_3 \cdot 6H_2O$ (pH = 6.0) at oxidizing (0.05 V vs. Ag/AgCl) and reducing (-0.45 V vs. Ag/AgCl) dc potentials with an ac potential of 10 mV on a mechanically polished Au working electrode that was irradiated with 2.45 W/cm^2 of 532 nm laser light. A graphite rod was used as the counter electrode.

Trial	R_S (Ω)		R_{CT} (Ω)		C_{dl} (nF)	
	0.05 V	-0.45 V	0.05 V	-0.45 V	0.05 V	-0.45 V
1	5.1	5.0	122.7	121.7	10.0	10.1
2	5.1	5.0	121.8	121.5	10.1	10.1
3	6.4	5.4	124.1	124.0	10.4	10.4
Avg.	5.5	5.1	122.9	122.4	10.2	10.2
Std. dev.	0.6	0.2	0.9	1.1	0.2	0.1

Table S6. Solution resistance (R_S), charge transfer resistance (R_{CT}), and double layer capacitance (C_{dl}) determined by fitting electrochemical impedance spectroscopy data to a simple Randles equivalent circuit. Measurements were made in an electrolytic solution containing 0.1 M $Na_2HPO_4 \cdot 7H_2O$ and 5 mM $Ru(NH_3)_6Cl_3 \cdot 6H_2O$ (pH = 6.0) at oxidizing (0.05 V vs. Ag/AgCl) and reducing (-0.45 V vs. Ag/AgCl) dc potentials with an ac potential of 10 mV on an electrochemically roughened Au working electrode that was irradiated with 2.45 W/cm^2 of 532 nm laser light. A graphite rod was used as the counter electrode.

Trial	R_S (Ω)		R_{CT} (Ω)		C_{dl} (nF)	
	0.05 V	-0.45 V	0.05 V	-0.45 V	0.05 V	-0.45 V
1	6.3	5.7	124.4	121.7	10.6	10.8
2	6.1	6.1	107.4	107.4	12.9	12.4
3	4.2	4.1	104.3	110.7	11.6	11.6
Avg.	5.5	5.3	112.0	113.3	11.7	11.6
Std. dev.	0.9	0.9	8.8	6.1	0.9	0.7

Table S7. Solution resistance (R_S), charge transfer resistance (R_{CT}), and double layer capacitance (C_{dl}) determined by fitting electrochemical impedance spectroscopy data to a simple Randles equivalent circuit. Measurements were made in an electrolytic solution containing 0.1 M $Na_2HPO_4 \cdot 7H_2O$ and 5 mM $Ru(NH_3)_6Cl_3 \cdot 6H_2O$ (pH = 6.0) at oxidizing (0.05 V vs. Ag/AgCl) and reducing (-0.45 V vs. Ag/AgCl) dc potentials with an ac potential of 10 mV on a mechanically polished Au working electrode that was irradiated with 2.45 W/cm^2 of 473 nm laser light. A graphite rod was used as the counter electrode.

Trial	R_S (Ω)		R_{CT} (Ω)		C_{dl} (nF)	
	**0.05 V**	**-0.45 V**	**0.05 V**	**-0.45 V**	**0.05 V**	**-0.45 V**
**1**	6.3	6.3	136.9	133.0	9.5	9.6
**2**	6.2	6.1	135.3	132.3	9.6	9.6
**3**	6.3	6.2	138.0	136.3	9.5	9.4
**Avg.**	6.3	6.2	136.7	133.9	9.5	9.5
**Std. dev.**	0.1	0.1	1.1	1.7	0.1	0.1

Table S8. Solution resistance (R_S), charge transfer resistance (R_{CT}), and double layer capacitance (C_{dl}) determined by fitting electrochemical impedance spectroscopy data to a simple Randles equivalent circuit. Measurements were made in an electrolytic solution containing 0.1 M $Na_2HPO_4 \cdot 7H_2O$ and 5 mM $Ru(NH_3)_6Cl_3 \cdot 6H_2O$ (pH = 6.0) at oxidizing (0.05 V vs. Ag/AgCl) and reducing (-0.45 V vs. Ag/AgCl) dc potentials with an ac potential of 10 mV on an electrochemically roughened Au working electrode that was irradiated with 2.45 W/cm^2 of 473 nm laser light. A graphite rod was used as the counter electrode.

Trial	R_S (Ω)		R_{CT} (Ω)		C_{dl} (nF)	
	**0.05 V**	**-0.45 V**	**0.05 V**	**-0.45 V**	**0.05 V**	**-0.45 V**
**1**	6.1	6.0	132.0	133.0	9.3	10.8
**2**	6.5	6.1	142.8	139.8	9.3	9.3
**3**	6.4	6.2	138.5	135.9	9.4	9.6
**Avg.**	6.3	6.1	137.8	136.2	9.3	9.9
**Std. dev.**	0.2	0.1	4.4	2.8	0.1	0.6

Table S9. Solution resistance (R_S), charge transfer resistance (R_{CT}), and double layer capacitance (C_{dl}) determined by fitting electrochemical impedance spectroscopy data to a simple Randles equivalent circuit. Measurements were made in an electrolytic solution containing 0.1 M $Na_2HPO_4 \cdot 7H_2O$ and 5 mM $Ru(NH_3)_6Cl_3 \cdot 6H_2O$ (pH = 6.0) at oxidizing (0.05 V vs. Ag/AgCl) and reducing (–0.45 V vs. Ag/AgCl) dc potentials with an ac potential of 10 mV on an electrochemically roughened Au working electrode in dark conditions with the electrode surface temperature set at 23.0, 24.2, 27.2, and 30.2 °C. A graphite rod was used as the counter electrode.

$T_{surface}$ (°C)	Trial	R_S (Ω)		R_{CT} (Ω)		C_{dl} (nF)	
		0.05 V	-0.45 V	0.05 V	-0.45 V	0.05 V	-0.45 V
23.0	1	3.9	3.3	152.8	155.2	6.6	6.6
	2	5.1	5.0	144.5	143.8	7.6	7.6
	3	3.8	3.8	158.9	159.4	8.0	8.0
	Avg.	4.3	4.0	152.1	152.8	7.4	7.4
	Std. dev.	0.6	0.7	5.9	6.6	0.6	0.6
24.2	1	3.1	3.2	154.7	153.6	6.7	6.7
	2	5.0	4.3	144.8	146.3	7.6	7.6
	3	4.5	3.7	154.7	155.5	8.0	8.0
	Avg.	4.2	3.7	151.4	151.8	7.4	7.4
	Std. dev.	0.8	0.4	4.7	4.0	0.5	0.5
27.2	1	3.0	3.1	151.4	150.7	6.9	6.9
	2	4.4	4.1	145.6	145.2	7.7	7.7
	3	3.7	3.5	153.2	153.1	8.1	8.1
	Avg.	3.7	3.6	150.1	149.7	7.6	7.6
	Std. dev.	0.6	0.4	3.2	3.3	0.5	0.5
30.2	1	3.2	3.2	150.1	149.1	6.9	7.0
	2	4.3	5.0	144.8	141.4	7.7	7.7
	3	4.0	4.0	148.4	147.0	8.1	8.2
	Avg.	3.8	4.1	147.8	145.8	7.6	7.6
	Std. dev.	0.5	0.7	2.2	3.2	0.5	0.5

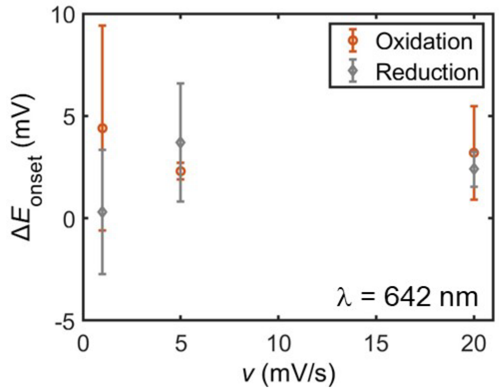

Figure S10. Change in the onset potential (E_{onset}) vs. Ag/AgCl (3 M KCl) for the oxidation (orange data) and reduction (gray data) of the $Ru(NH_3)_6^{3+/2+}$ redox probe as a function of scan rate when electrochemically roughened Au electrodes were irradiated with 2.45 W/cm^2 of 642 nm laser light and in dark conditions. $\Delta E_{onset} = E_{onset,\ light\ irradiation} - E_{onset,\ dark\ conditions}$. Data points are average values from three independent trials and the errors bars represent the standard deviations of the measurements.

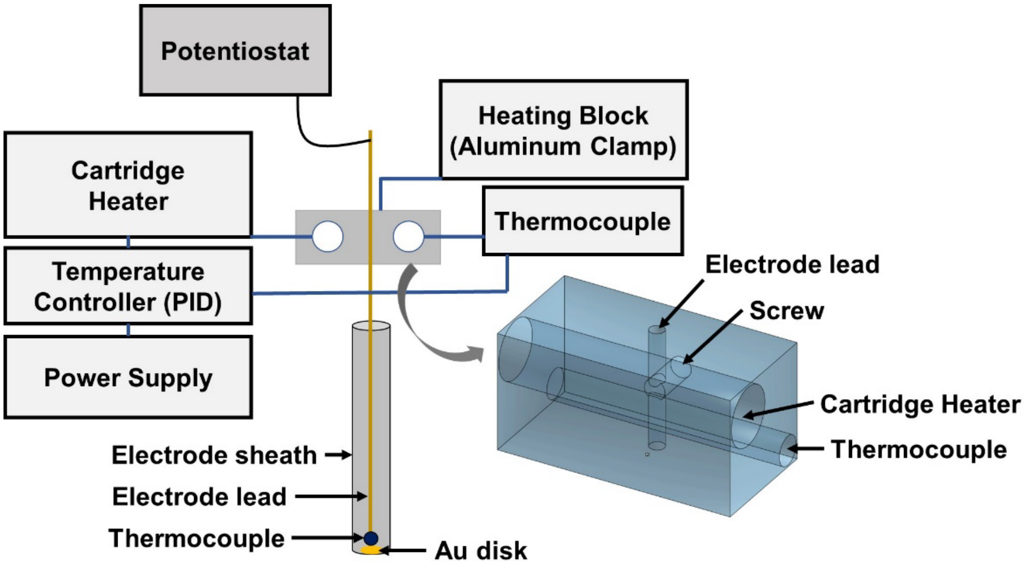

Figure S11. Schematic of system used to heat and measure the surface temperature of a Au disk working electrode.

Supplementary Methods 1

Simulations for heat conduction across Au disk electrode

Finite-element simulations of heat conduction were performed using COMSOL Multiphysics, version 6.1. The geometry of the system under study was designed as a 2D axisymmetric model, consisting of two concentric cylinders formed by the complete revolution of the plane depicted in Figure S12A. In this configuration, the inner cylinder is the working electrode assembly, while the outer one represents the electrolyte. The dimensions of the electrolyte domain are characterized by the radius of the electrochemical cell (R_{cell} = 14 mm) and the height of the liquid level (H = 24 mm). The electrode assembly consists of an inert shaft immersed 14 mm into the electrolyte (thus 10 mm from the bottom of the cell), and a radius of 2 mm. The Au disk is a cylinder with dimensions $R_{\mathrm{electrode}}$ = 1 mm and $H_{\mathrm{electrode}}$ = 2.5 mm. The polymer sheath was modeled as PTFE, as a close approximation of Kel-F. The finite elements were tessellated into a physics-controlled mesh using the "Normal" element size preset, shown in Figure S12B.

The energy balance, shown in Equation 1, is solved for both solids,

$$\rho C_p \left(\frac{\partial T}{\partial t} + \vec{u}_{\mathrm{trans}} \cdot \nabla T\right) + \nabla \cdot (\vec{q} + \vec{q}_r) = -\alpha T{:}\frac{dS}{dt} + Q \tag{1}$$

where $\vec{u}_{\mathrm{trans}}$ is the translational motion velocity, $\vec{q}$ is the heat flux by conduction, $\vec{q}_r$ is the heat flux by radiation, α is the material's thermal expansion coefficient and S is the second Piola-Kirchhoff stress tensor. Assuming the system reached steady-state conditions ($d/dt = 0$) and heat transfer by radiation is negligible when compared to conduction, Equation 2 is obtained.

$$\rho C_p \vec{u}_{\mathrm{trans}} \cdot \nabla T = -\nabla \cdot \vec{q} \tag{2}$$

The heat flux due to conduction is given by Equation 3, where k is the thermal conductivity of the material.

$$\vec{q} = -k\nabla T \tag{3}$$

The Heat Transfer in Liquids and Solids interface in COMSOL was used to calculate the temperature distribution across the Au disk when the electrode-electrolyte interface boundary was set to a temperature 24.2, 27.2, or 30.2 °C (Figure S12C, D).

Table S10. The properties used for each material used to simulate heat conduction across the Au disk electrode.

Material	ρ (kg m^{-3})	k (W m^{-1} K^{-1})	C_p (J kg^{-1} K^{-1})
Au	1.90×10^4	317	130
PTFE*	2.2×10^3	0.318	850

*Surrogate for Kel-F.

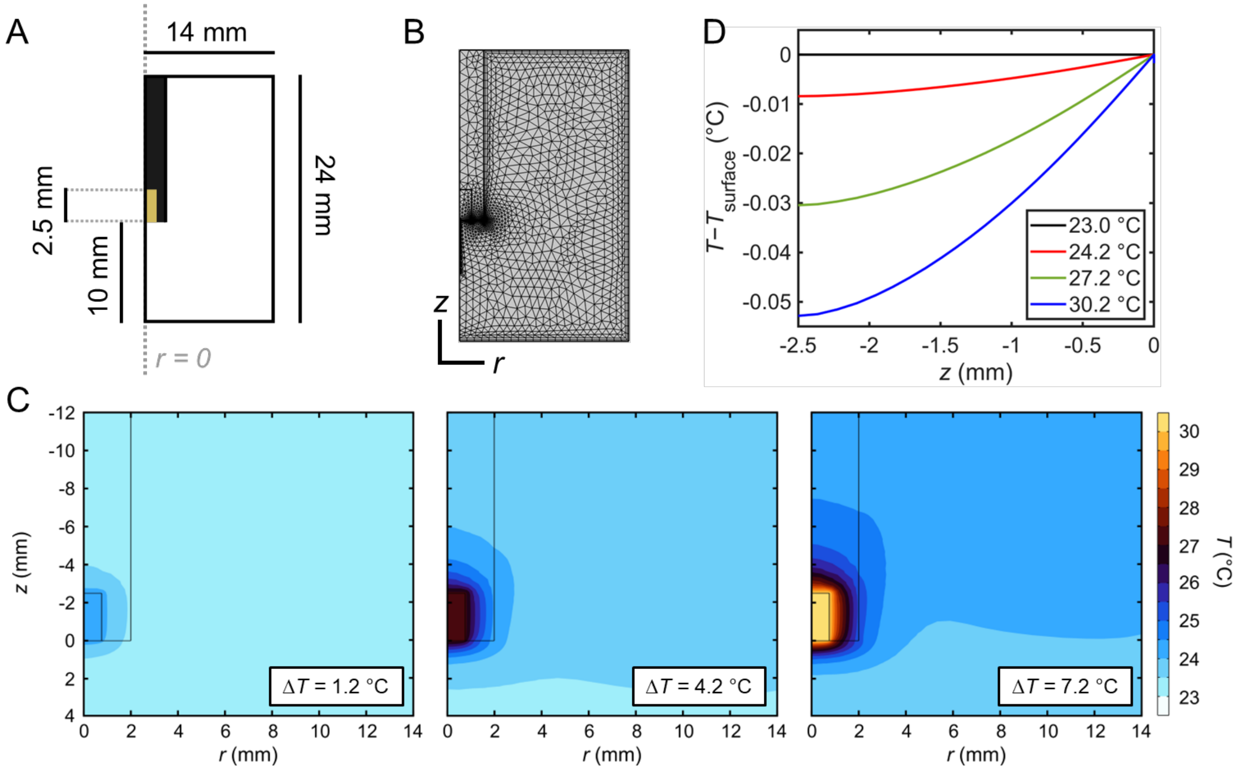

Figure S12. (A) Geometry and (B) finite element mesh used for simulations of heat conduction across the Au disk electrode. (C) Simulated temperature map resulting from the electrode surface at the electrode-electrolyte interface being heated to a temperature of 24.2, 27.2, or 30.2 °C (ΔT of 1.2, 4.2, 7.2 °C, respectively). (D) Difference in temperature across the Au disk electrode when the electrode-electrolyte interface is heated to a temperature of 23.0, 24.2, 27.2, or 30.2 °C.

Table S11. Bulk electrolyte and electrode surface temperature (°C) measured in an electrolytic solution containing 0.1 M $Na_2HPO_4 \cdot 7H_2O$ and 5 mM $Ru(NH_3)_6Cl_3 \cdot 6H_2O$ (pH = 6.0) when an electrochemically roughened Au electrode was irradiated with 2.45 W/cm^2 of 473 nm laser light, 2.45 W/cm^2 of 532 nm laser light, or 2.45 W/cm^2 of 642 nm laser light.

**Trial**	**Dark**		**473 nm**		**532 nm**		**642 nm**	
	$T_{Surface}$	T_{Bulk}	$T_{Surface}$	T_{Bulk}	$T_{Surface}$	T_{Bulk}	$T_{Surface}$	T_{Bulk}
**1**	23.0	23.0	30.2	23.0	27.1	23.0	24.1	23.0
**2**	23.0	23.0	30.1	23.0	27.3	23.0	24.2	23.0
**3**	23.0	23.0	30.3	23.0	27.2	23.0	24.3	23.0
**Avg.**	23.0	23.0	30.2	23.0	27.2	23.0	24.2	23.0
**Std. dev.**	0.0	0.0	0.1	0	0.1	0	0.1	0

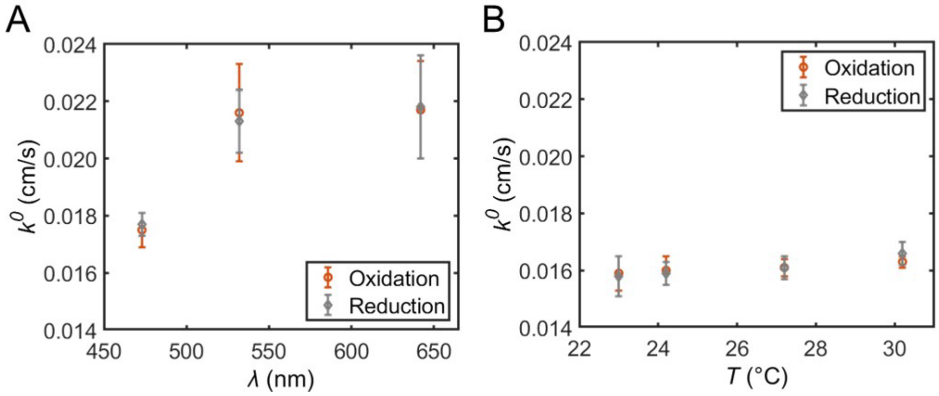

Figure S13. Heterogeneous electron transfer rate constants (k^0) plotted as a function of (A) excitation wavelength and (B) set electrode surface temperature in dark conditions. The intensity of light excitation was fixed across all wavelengths at 2.45 W/cm^2. Data points are average values determined from three independent experimental trials and the errors bars represent the standard error of k^0 determinations.

Table S12. Heterogeneous electron transfer rate constants (k^0) of the $Ru(NH_3)_6^{3+/2+}$ redox probe determined from electrochemical impedance spectroscopy.

	**k^0 (cm/s) oxidation**	**k^0 (cm/s) reduction**
**2.45 W/cm^2, 473 nm light**	0.0175 ± 0.0006	0.0177 ± 0.0004
**2.45 W/cm^2, 532 nm light**	0.0216 ± 0.0017	0.0213 ± 0.0012
**2.45 W/cm^2, 642 nm light**	0.0217 ± 0.0017	0.0218 ± 0.0018
**Dark, $T_{surface}$ = 23.0 °C**	0.0159 ± 0.0006	0.0158 ± 0.0007
**Dark, $T_{surface}$ = 24.2 °C**	0.0160 ± 0.0005	0.0159 ± 0.0004
**Dark, $T_{surface}$ = 27.2 °C**	0.0161 ± 0.0003	0.0161 ± 0.0004
**Dark, $T_{surface}$ = 30.2 °C**	0.0163 ± 0.0002	0.0166 ± 0.0004

Supplementary Note 2.

Heterogeneous electron transfer rate constants (k^0) were determined using Equation 4,

$$k^0 = \frac{RT}{n^2F^2ACR_{CT}} \quad (4)$$

where R is the gas constant (J/mol·K), T is temperature (K), n is the number of electrons transferred, F is Faraday's constant (C/mol), A is the electrode surface area (cm^2), C is concentration (mol/cm^3), and R_{CT} is the charge transfer resistance (Ω) determined from electrochemical impedance spectroscopy.

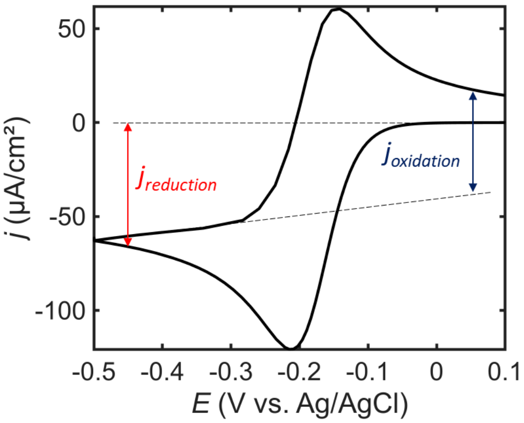

Figure S14. Schematic illustrating how the reduction and oxidation current densities are tabulated in the mass transport-limiting regimes of –0.45 V and 0.05 V, respectively.

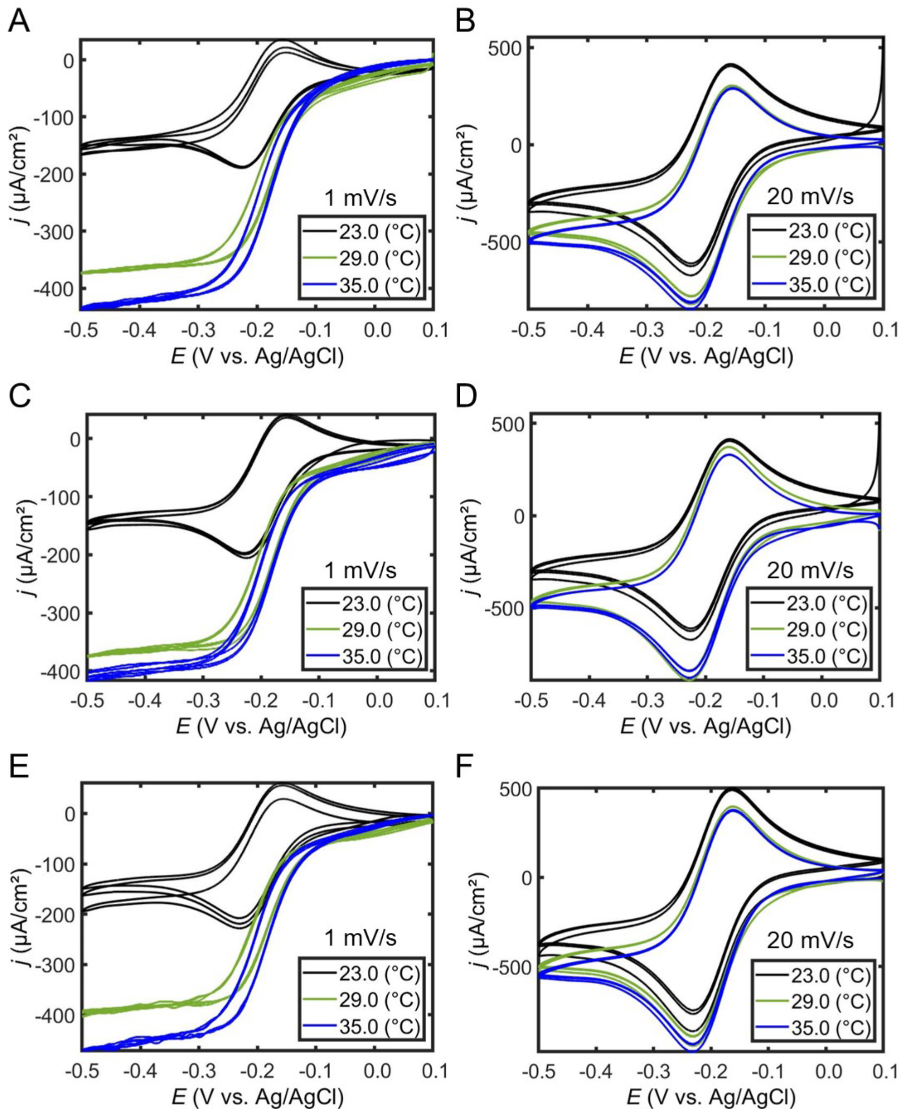

Figure S15. Cyclic voltammograms measured at electrochemically roughened Au working electrodes in an electrolytic solution containing 0.1 M $Na_2HPO_4{\cdot}7H_2O$ and 5 mM $Ru(NH_3)_6Cl_3{\cdot}6H_2O$ (pH = 6.0) with a scan rate of (A) 1 mV/s and (B) 20 mV/s. (C-D) and (E-F) Repeat trials of panels A-B on separately prepared Au electrodes. Data were acquired in dark conditions with the electrode surface set at different temperatures as indicated in the legends. A graphite rod was used as the counter electrode.

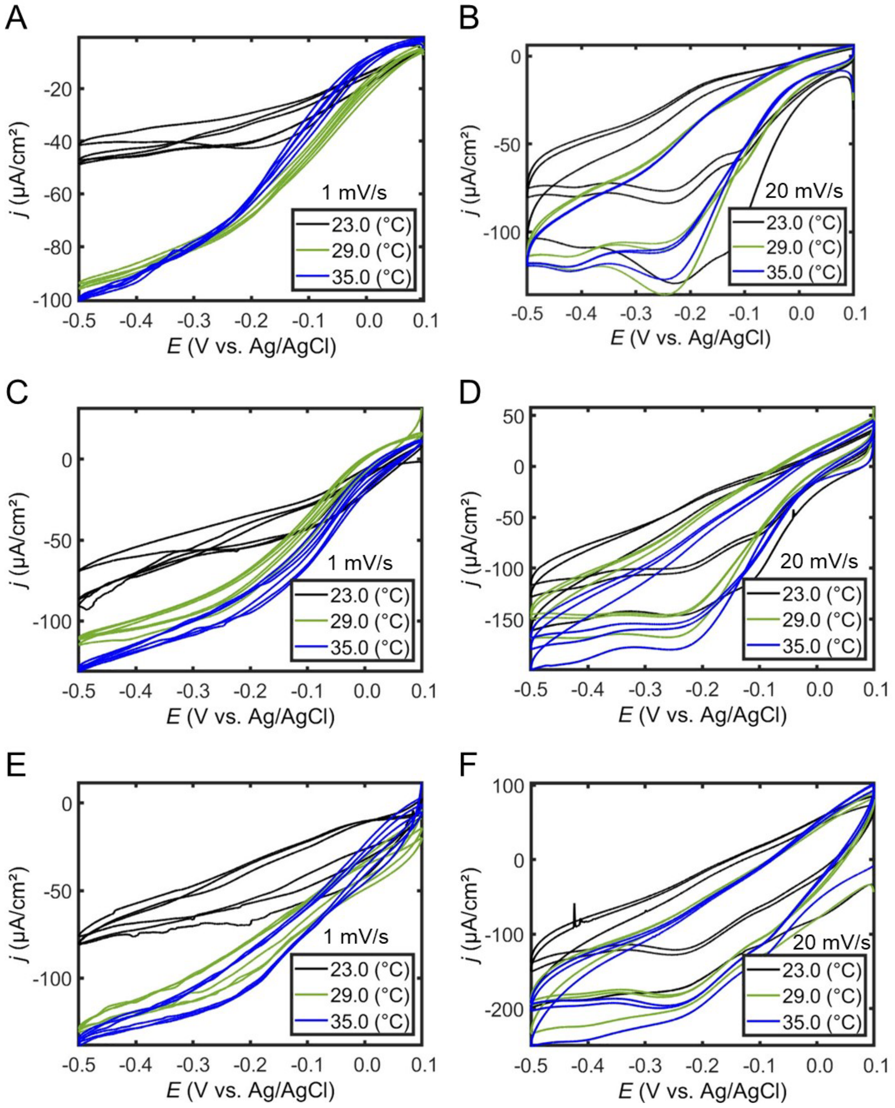

Figure S16. Cyclic voltammograms measured at electrochemically roughened Au working electrodes in an electrolytic solution containing 0.1 M $Na_2HPO_4 \cdot 7H_2O$ (pH = 6.0) with a scan rate of (A) 1 mV/s and (B) 20 mV/s. (C-D) and (E-F) Repeat trials of panels A-B on separately prepared Au electrodes. Data were acquired in dark conditions with the electrode surface set at different temperatures as indicated in the legends. A graphite rod was used as the counter electrode.

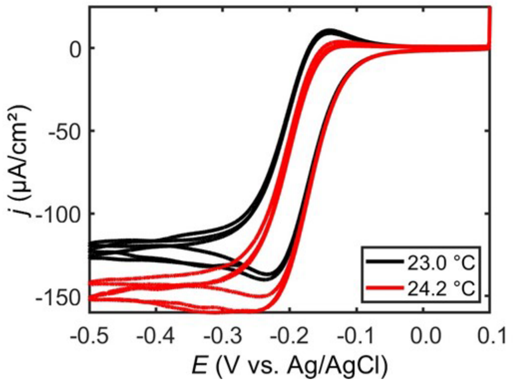

Figure S17. Cyclic voltammograms measured at electrochemically roughened Au working electrodes in an electrolytic solution containing 0.1 M $Na_2HPO_4 \cdot 7H_2O$ and 5 mM $Ru(NH_3)_6Cl_3 \cdot 6H_2O$ (pH = 6.0) with a scan rate of 1 mV/s. Data were acquired in dark conditions with the bulk electrolytic solution temperature set at 23.0 °C (black curves) and 24.2 °C (red curves). A graphite rod was used as the counter electrode.

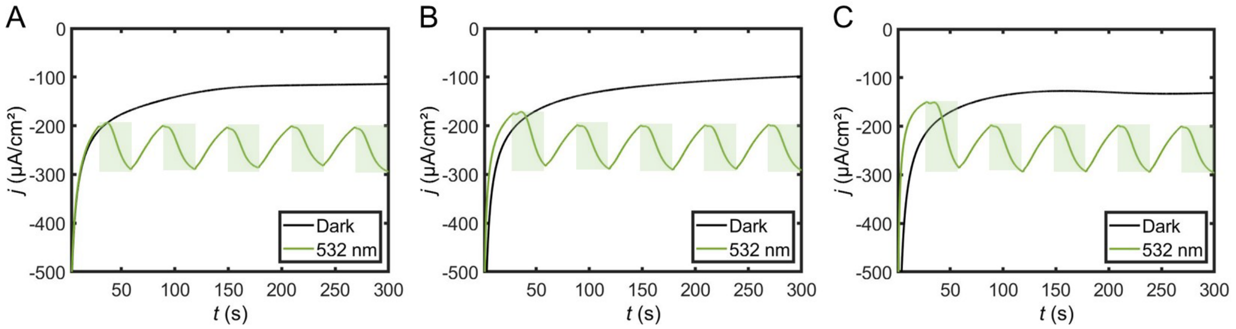

Figure S18. Chronoamperograms acquired by setting the applied potential of an electrochemically roughened Au disk electrode to –0.45 V vs. Ag/AgCl (3 M KCl) in an electrolytic solution containing 0.1 M $Na_2HPO_4 \cdot 7H_2O$ and 5 mM $Ru(NH_3)_6Cl_3 \cdot 6H_2O$ (pH = 6.0). Dark curves were acquired under dark conditions and green curves were acquired under chopped illumination. Green shaded areas indicate the times when the electrode was irradiated with 2.45 W/cm^2 of 532 nm laser light. A, B, and C are repeat trails on separately prepared electrodes.

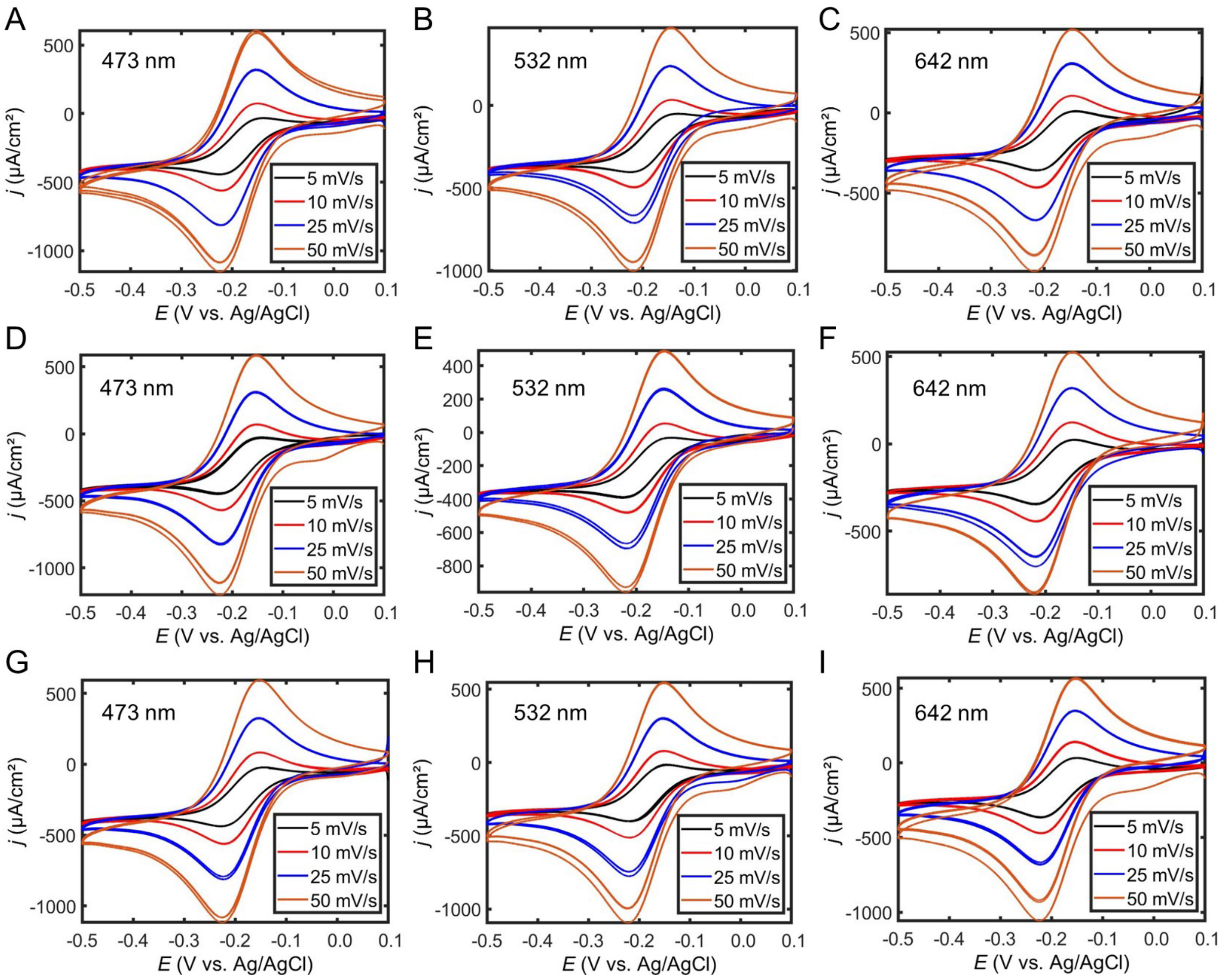

Figure S19. Cyclic voltammograms measured at electrochemically roughened Au working electrodes in an electrolytic solution containing 0.1 M $Na_2HPO_4 \cdot 7H_2O$ and 5 mM $Ru(NH_3)_6Cl_3 \cdot 6H_2O$ (pH = 6.0) at different scan rates as indicated in the legends when the electrode was irradiated with 2.45 W/cm^2 of (A) 473 nm, (B) 532 nm, and (C) 642 nm laser light. (D-F) and (G-I) Repeat trials of panels A-C on separately prepared Au electrodes. A graphite rod was used as the counter electrode.

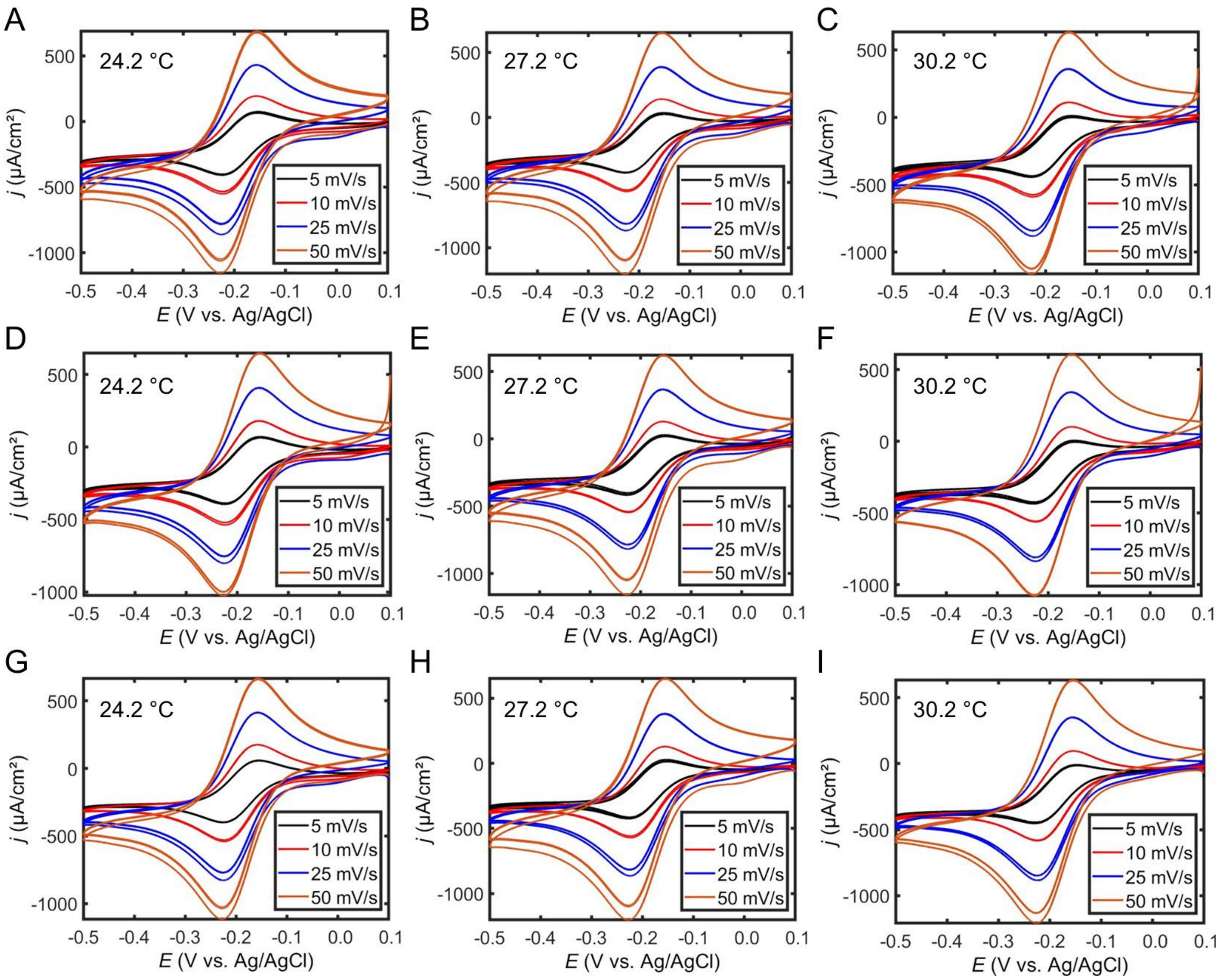

Figure S20. Cyclic voltammograms measured at electrochemically roughened Au working electrodes in an electrolytic solution containing 0.1 M $Na_2HPO_4 \cdot 7H_2O$ and 5 mM $Ru(NH_3)_6Cl_3 \cdot 6H_2O$ (pH = 6.0) at different scan rates as indicated in the legends in dark conditions with the electrode surface temperature set at (A) 24.2 °C, (B) 27.2 °C, and (C) 30.2 °C. (D-F) and (G-I) Repeat trials of panels A-C on separately prepared Au electrodes. A graphite rod was used as the counter electrode.

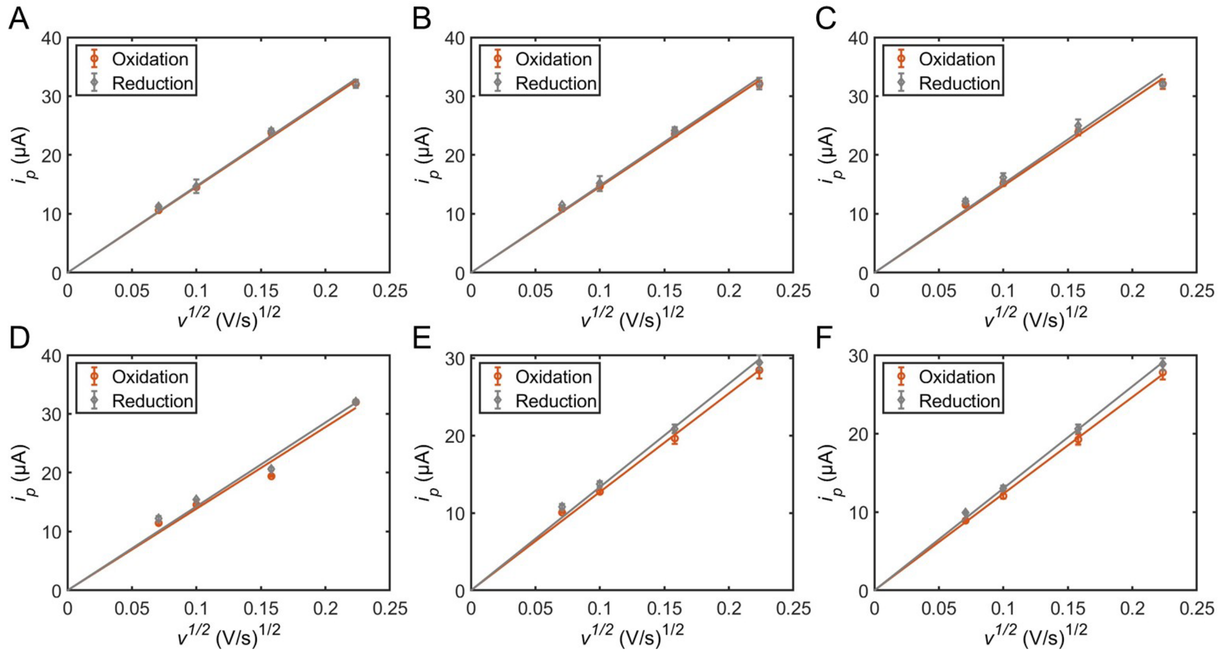

Figure S21. Anodic (orange data) and cathodic (gray data) peak currents (i_p) obtained from cyclic voltammograms measured at electrochemically roughened Au disk working electrodes in an electrolytic solution containing 0.1 M $Na_2HPO_4 \cdot 7H_2O$ and 5 mM $Ru(NH_3)_6Cl_3 \cdot 6H_2O$ (pH = 6.0) as a function of the square root of the scan rate (v). Data were acquired in dark conditions with the electrode surface temperature set at (A) 24.2 °C, (B) 27.2 °C, (C) 30.2 °C and when the electrode was irradiated with 2.45 W/cm^2 of (D) 473 nm, (E) 532 nm, (F) 642 nm laser light. Data points are average values from three independent trials and the errors bars represent the standard deviations of the measurements. Solid lines represent linear fits to the data.

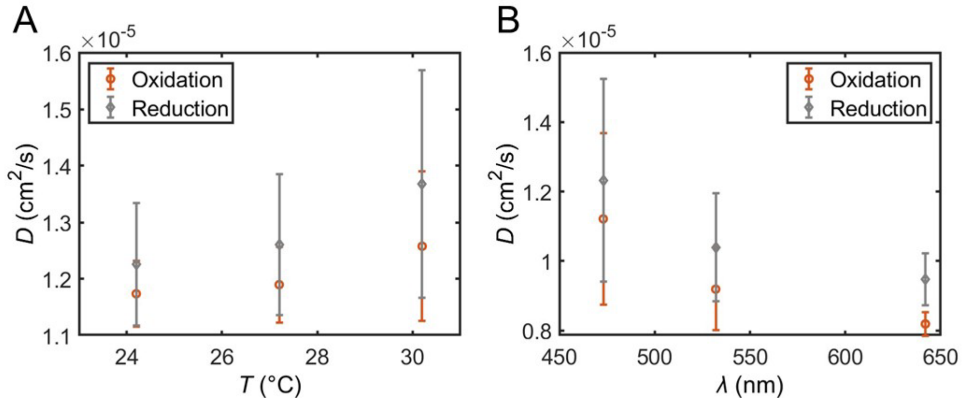

Figure S22. Diffusion coefficients (*D*) determined using the anodic (orange data) and cathodic (gray data) peak currents and the Randles-Ševčík equation as a function of (A) set electrode surface temperature in dark conditions and (B) excitation light wavelength at a fixed intensity of 2.45 W/cm^2. Data points are average values from three independent trials and the errors bars represent the standard error of *D* determinations.

Supplementary Methods 2

Electrochemical simulation methods.

Finite elements simulation of the electrochemical system was performed using COMSOL Multiphysics, version 6.1. The system was modeled assuming the following redox reaction:

$$\mathrm{O} + e \rightleftharpoons \mathrm{R} \tag{5}$$

The reaction kinetics were modeled using the Butler-Volmer equation to calculate the local current density at the electrode surface as a function of the overpotential:

$$j = j_0\left[e^{\alpha n f \eta} - e^{-(1-\alpha) n f \eta}\right] \tag{6}$$

In which,

$$f = \frac{F}{RT} \tag{7}$$

$$\eta = E - E_{\mathrm{eq}} \tag{8}$$

$$E = \phi_s - \phi_l \tag{9}$$

where j is the current density at the electrode surface, j_0 is the exchange current density, α is the anodic transfer coefficient (estimated as 0.5), n is the stoichiometric number of exchanged electrons (1, according to Equation 5), E is the electrode potential, ϕ_s is the electric potential of the electrode, ϕ_l is the electrolyte potential, E_{eq} is the equilibrium potential, F is Faraday's constant (96487 C/mol), R is the ideal gas constant (8.3145 J/mol·K), and T is the temperature. The exchange current density j_0 is calculated with the mass action law and a reference exchange current density value, $j_{0,\mathrm{ref}}$, which was estimated through Equation 10,

$$j_{0,\mathrm{ref}} = k^0 F c_{\mathrm{ref}} \tag{10}$$

where k^0 is the heterogeneous electron transfer rate constant (determined experimentally as 0.021 cm/s) and c_{ref} is the reference concentration, defined as 1 M.

The electric potential ϕ_s is known as a function of time, determined by the cyclic voltammetry waveform, while E_{eq} is defined by the Nernst equation (11) and determines the concentration of both species at the electrode surface.

$$E_{\mathrm{eq}} = E_{\mathrm{eq}}^0 - \frac{1}{nf}\ln\frac{c_{\mathrm{R}}}{c_{\mathrm{O}}} \tag{11}$$

The transport of O and R in the electrolyte was modeled using a mass balance and the Nernst-Planck equation (12),

$$\frac{\partial c_i}{\partial t} = \nabla \cdot \left[(D_i \nabla c_i) + z_i u_{\mathrm{m},i} F c_i \nabla \phi_l - \vec{u} c_i\right] + R_i \tag{12}$$

where D_i is the diffusion coefficient of species i (assumed as 0.5×10^{-5} cm^2/s for both species based

on experimental data), z_i is the charge of species i, $\vec{u}$ is the velocity field of the fluid, and R_i represents the reaction rate of species i, which is a function of the current density j. The velocity field $\vec{u}$ determines the flow of the bulk fluid which, in turn, is governed by the Navier-Stokes equations. For weakly compressible flow, the continuity equation (13) is:

$$\frac{\partial \rho}{\partial t} + \nabla \cdot (\rho \vec{u}) = 0 \tag{13}$$

And the momentum equation (14) is,

$$\rho \frac{\partial \vec{u}}{\partial t} + \rho(\vec{u} \cdot \nabla)\vec{u} = \nabla \cdot \left[-pI + \mu(\nabla \vec{u} + (\nabla \vec{u})^{\mathrm{T}}) - \frac{2}{3}\mu(\nabla \cdot \vec{u})I \right] + \vec{F} + (\rho - \rho_{\mathrm{ref}})\vec{g} \tag{14}$$

where ρ is the density of the fluid, p is the pressure, μ is the dynamic viscosity, $\vec{F}$ is the volume force vector, and $\vec{g}$ is the acceleration vector due to gravity. In this model, since the force volume $\vec{F}$ and the initial velocity field $\vec{u}$ are assumed to be null, the driving force for convection is a change in ρ, which is caused by temperature gradients. The temperature is determined by solving the heat balance expressed by Equation 15,

$$\rho C_p \left(\frac{\partial T}{\partial t} + \vec{u} \cdot \nabla T \right) + \nabla \cdot \vec{q} = Q_p + Q_{\mathrm{vd}} + Q \tag{15}$$

where C_p is the specific heat capacity at constant stress, $\vec{q}$ is the heat flux due to conduction, Q_p is work done by pressure changes, Q_{vd} is viscous dissipation and Q represents any other heat sources.

The geometry of the system under study was designed as a 2D axisymmetric model, consisting of two concentric cylinders formed by the complete revolution of the plane depicted in Figure S23A along the z-axis at $r = 0$. In this configuration, the inner cylinder is the working electrode assembly, while the outer one represents the electrolyte. The dimensions of the electrolyte domain are characterized by the radius of the electrochemical cell (R_{cell} = 14 mm) and the height of the liquid level (H = 24 mm). The electrode assembly consists of an inert shaft immersed 14 mm into the electrolyte (thus 10 mm from the bottom of the cell), and a radius of 2 mm. The electrode surface, which is the Au disk ($R_{\mathrm{electrode}}$ = 1 mm), is exposed to the electrolyte, as shown in Figure S23Aii, and is the origin of the z-axis. Due to this geometry, a 2D axisymmetric model represents the system as well as a 3D model at a fraction of the computational effort since the symmetry implies there are no gradients in the azimuthal direction ($\partial/\partial\varphi = 0$).

Equations 5–12 were computed in COMSOL by the Electroanalysis interface. The Nonisothermal Flow Multiphysics was used to couple the Laminar Flow interface (Equations 13 and 14) and the Heat Transfer in Fluids interface (Equation 15). The electrode assembly was assumed to be thermally and electrically insulated. Therefore, heat transfer due to photothermal heating and electron transfer would only take place at the electrode surface. In the heat transfer equation, Q_p and Q_{vd} were neglected since the system is open and viscous dissipation is low in the laminar

regime, respectively. It is assumed that there is no mass or charge transfer at the outer bounds of the electrolyte, but Q is assumed to be the heat flux from the electrolyte to ambient air by convection,

$$Q = -h(T - T_{\text{air}}) \tag{16}$$

where h is the convective heat transfer coefficient of air, assumed to be 20 W/m^2·K, and T_{air} is the ambient temperature, 23.0 °C. Furthermore, since the concentration of supporting electrolyte is much greater than those of O and R, it is reasonable to assume that there are no ohmic losses, and the electrolyte potential is insignificant, or $\phi_l = 0$. This also simplifies Equation 12 by eliminating the migration term, which is also negligible for O and R in the presence of supporting electrolyte.

The finite elements were tessellated into a physics-controlled mesh using the "Normal" element size preset, shown in Figure S23B.

Initial modeling of heat and mass transfer showed that the system reached steady-state conditions in ca. 40 s, which is much faster than the time scale of the cyclic voltammetry experiments. Moreover, experimentally, the electrode surface was heated by light irradiation several minutes prior to voltammetry measurements. Thus, Equations 13–15 were treated in their stationary forms, i.e., $\partial/\partial t = 0$. As a consequence, the study was performed in two steps: first, Equations 13–15 are solved for the specified electrode surface temperature and next, Equations 5–12 are solved using the steady-state velocity field $\vec{u}$ determined during the first step.

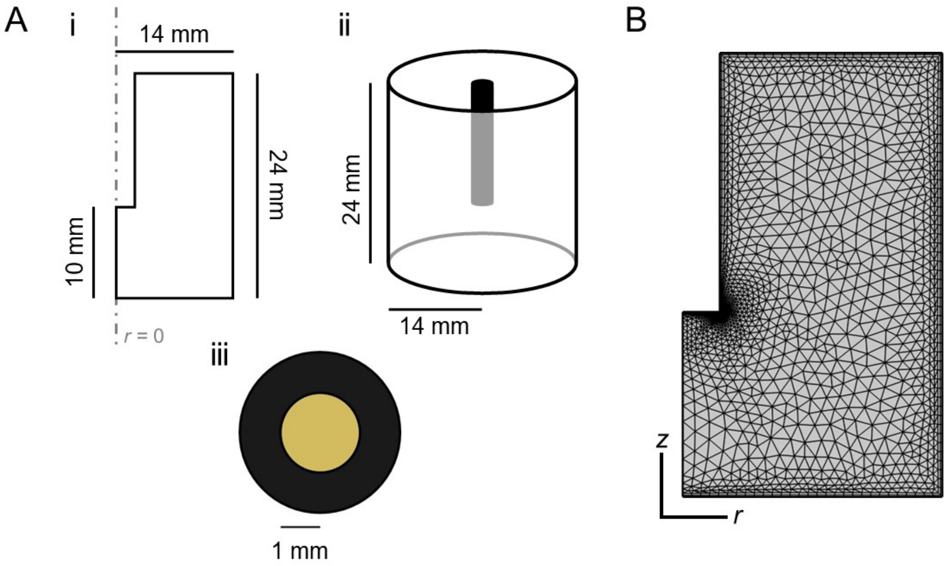

Figure S23. (A) Geometry and (B) finite element mesh used for simulations of cyclic voltammetry of a redox probe at a heated electrode surface. (i) two-dimensional and (ii) three-dimensional view (formed by revolution about the z axis) of the system. (iii) Electrode surface.

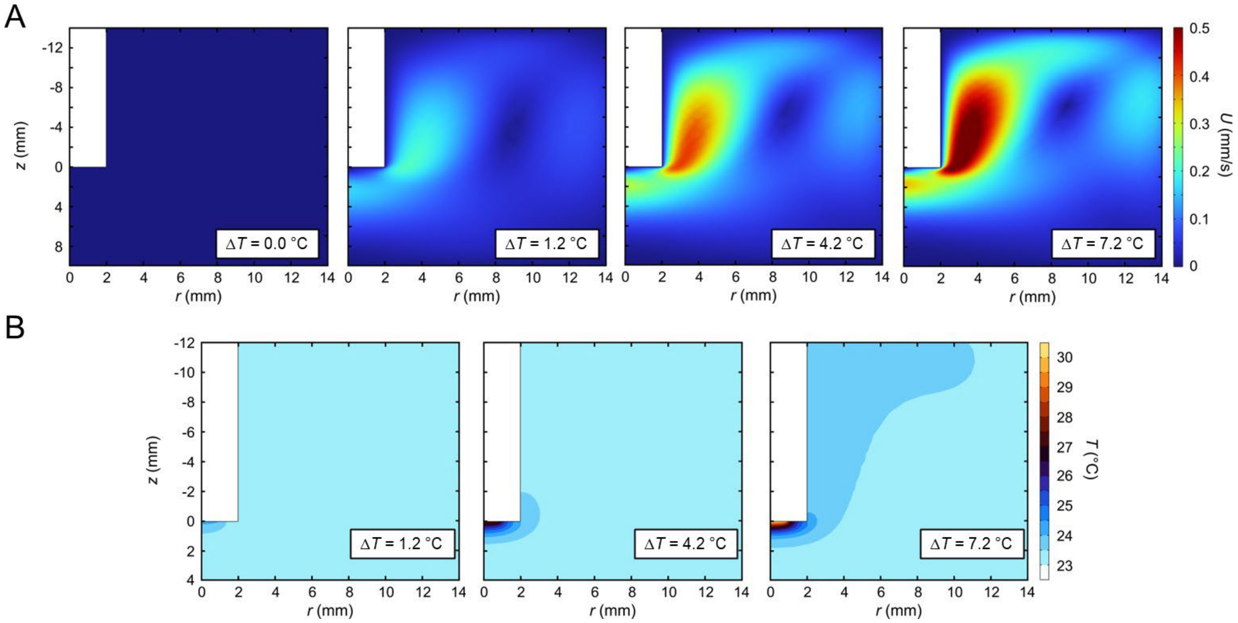

Figure S24. (A) Simulated fluid velocity (U) at different ΔT values. (B) Simulated temperature map resulting from a heated electrode surface. The bulk fluid was set to an initial temperature of 23.0 °C and the electrode surface was set to a temperature of 23.0, 24.2, 27.2, or 30.2 °C (ΔT of 0.0, 1.2, 4.2, 7.2 °C, respectively).

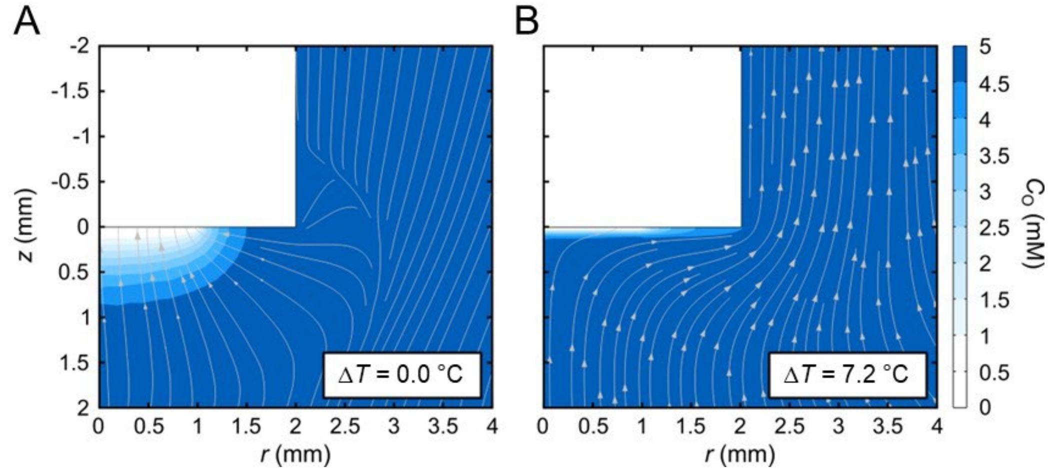

Figure S25. Maps of the concentration of the oxidized species (O) near the electrode surface in the mass-transport limited regime ($E = -0.45$ V, $v = 1$ mV/s) at (A) $\Delta T = 0.0$ °C and (B) $\Delta T = 7.2$ °C. The bulk fluid was set to an initial temperature of 23.0 °C and the electrode surface was set to a temperature of 23.0 or 30.2 °C (ΔT of 0.0 or 7.2 °C, respectively). Streamlines represent the direction of the mass flux of species O, while the magnitude of the arrows represents the relative scalar mass flux of species O.

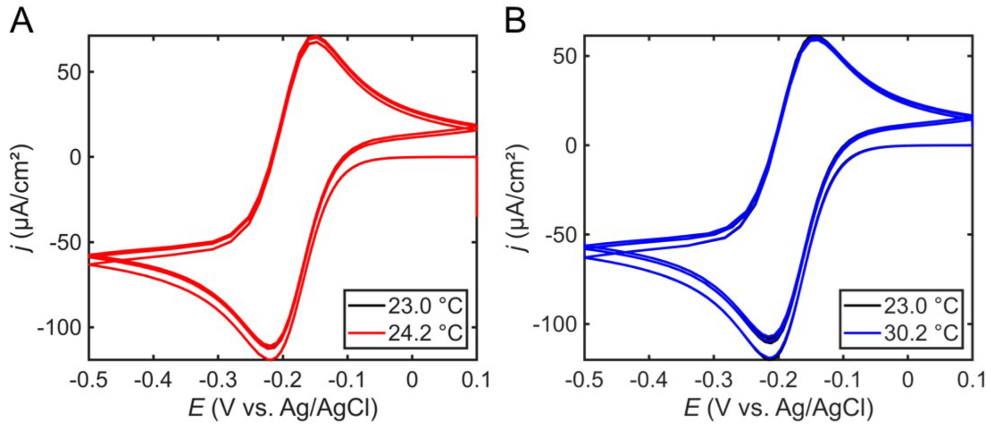

Figure S26. Cyclic voltammograms simulated in an aqueous solvent containing a reversible redox couple with characteristics of $Ru(NH_3)_6^{3+/2+}$ at a scan rate of 1 mV/s and a bulk fluid temperature of 23.0 °C (black curves), (A) 24.2 °C (red curves), or (B) 30.2 °C (blue curves). Three potential cycles were simulated for each condition. Cyclic voltammograms at the different bulk fluid temperatures are nearly identical resulting in overlapping curves.

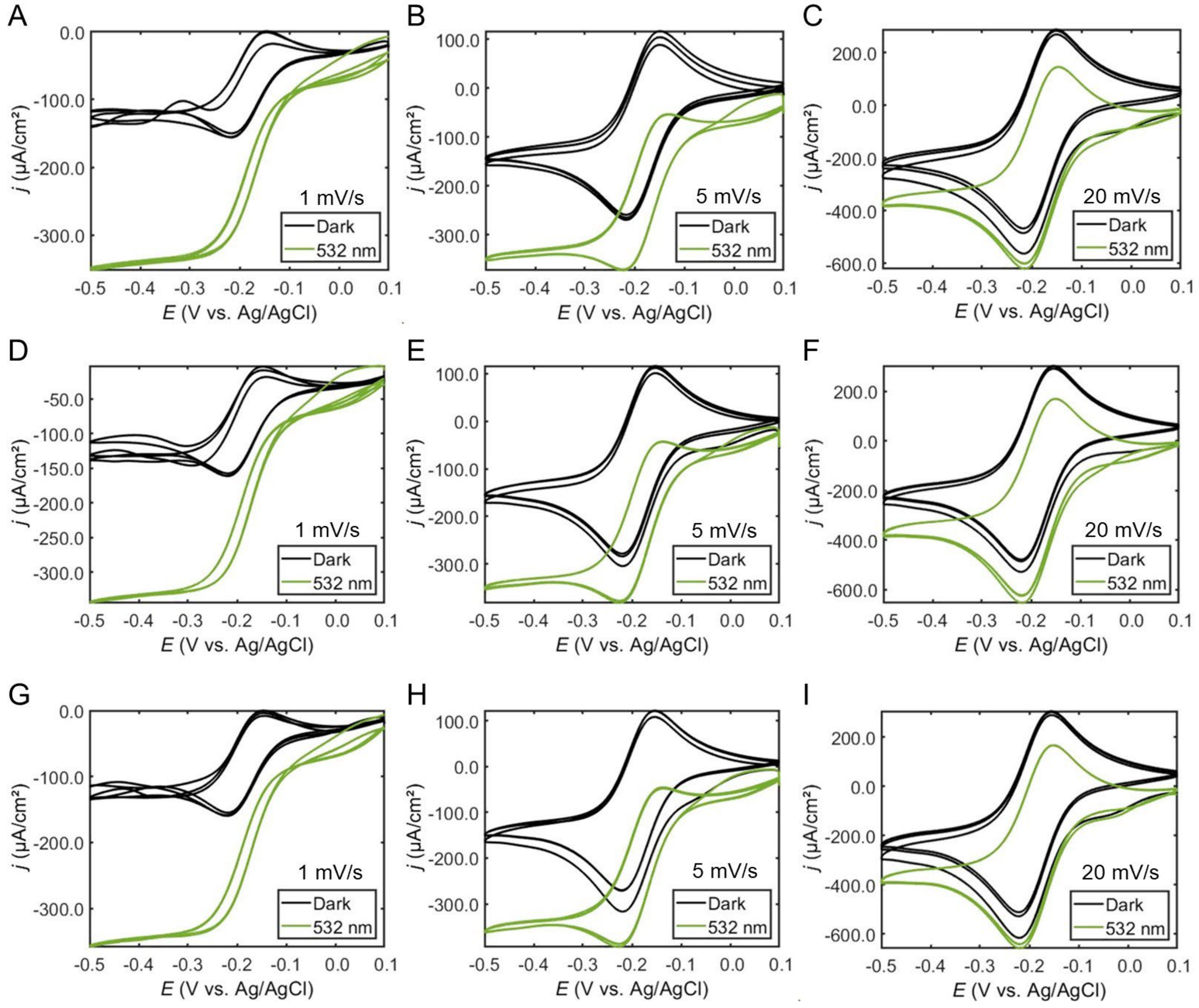

Figure S27. Cyclic voltammograms measured at electrochemically roughened Au working electrodes in an electrolytic solution containing 0.1 M $Na_2HPO_4 \cdot 7H_2O$ and 5 mM $Ru(NH_3)_6Cl_3 \cdot 6H_2O$ (pH = 6.0) with a scan rate of (A) 1 mV/s, (B) 5 mV/s, and (C) 20 mV/s. (D-F) and (G-I) Repeat trials of panels A-C on separately prepared Au electrodes. Black curves were obtained in dark conditions and green curves were obtained by irradiating Au electrodes with 2.45 W/cm^2 of 532 nm laser light. A graphite rod was used as the counter electrode.

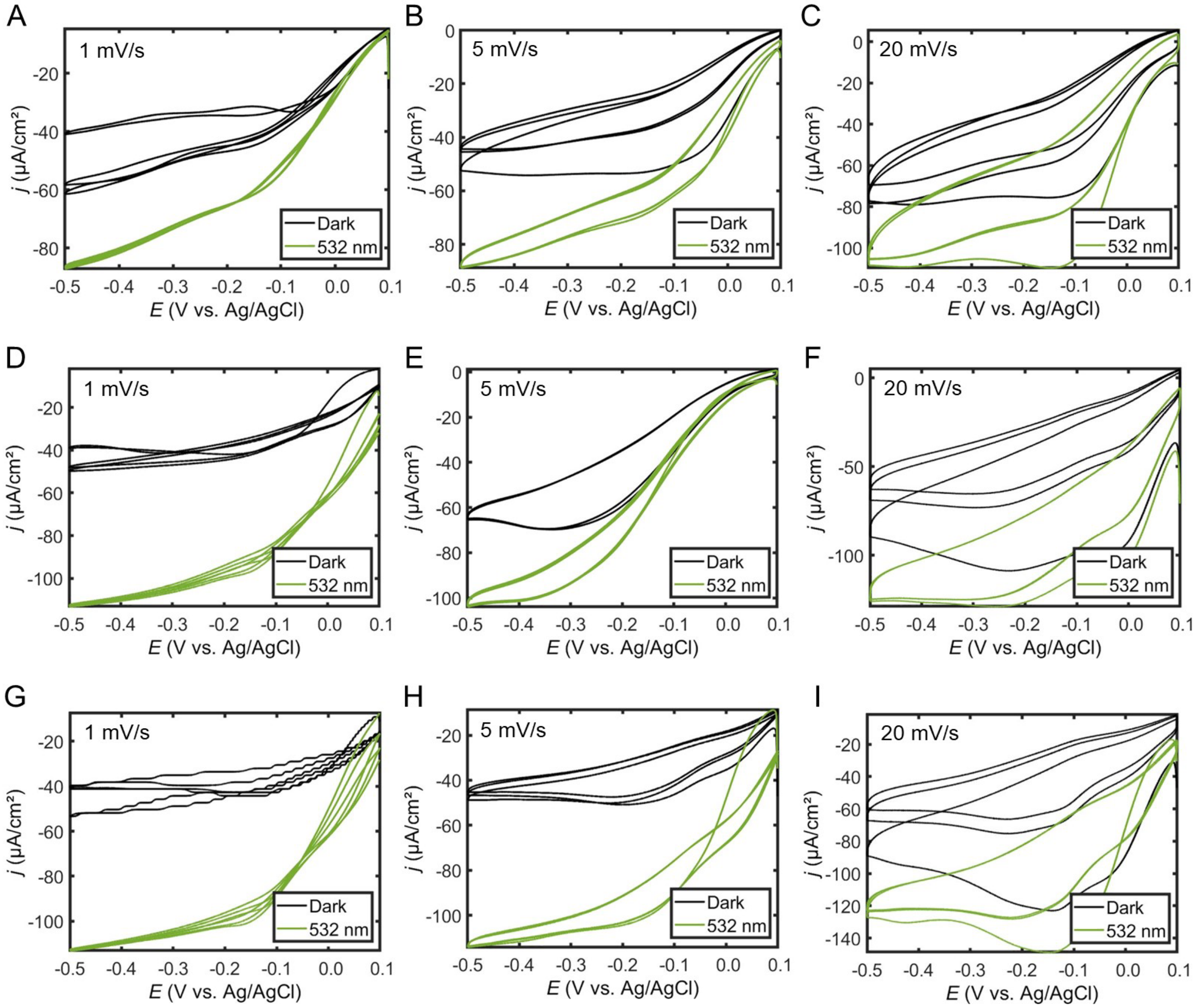

Figure S28. Cyclic voltammograms measured at electrochemically roughened Au working electrodes in an electrolytic solution containing 0.1 M $Na_2HPO_4 \cdot 7H_2O$ (pH = 6.0) with a scan rate of (A) 1 mV/s, (B) 5 mV/s, and (C) 20 mV/s. (D-F) and (G-I) Repeat trials of panels A-C on separately prepared Au electrodes. Black curves were obtained in dark conditions and green curves were obtained by irradiating Au electrodes with 2.45 W/cm^2 of 532 nm laser light. A graphite rod was used as the counter electrode.

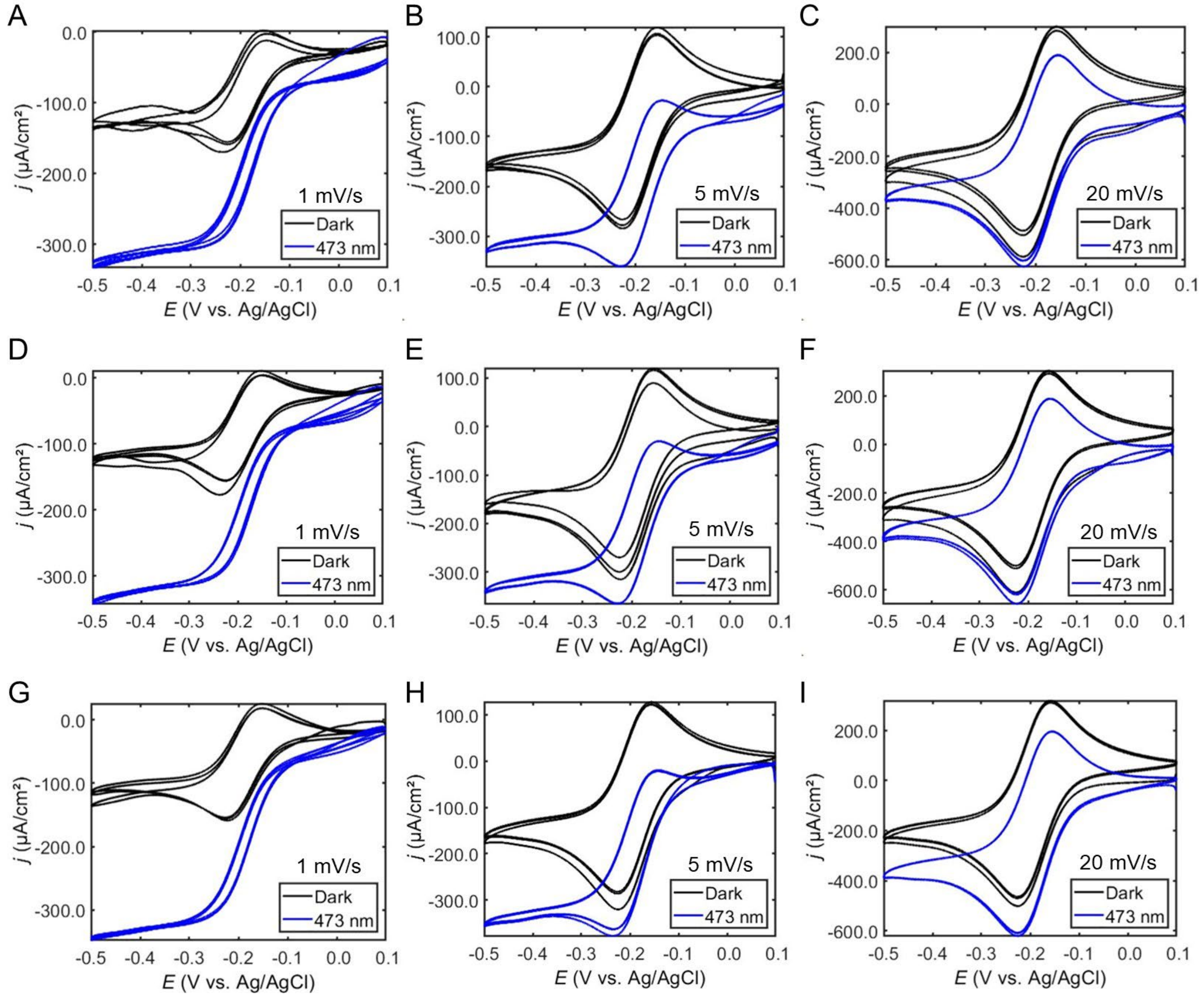

Figure S29. Cyclic voltammograms measured at electrochemically roughened Au working electrodes in an electrolytic solution containing 0.1 M $Na_2HPO_4 \cdot 7H_2O$ and 5 mM $Ru(NH_3)_6Cl_3 \cdot 6H_2O$ (pH = 6.0) with a scan rate of (A) 1 mV/s, (B) 5 mV/s, and (C) 20 mV/s. (D-F) and (G-I) Repeat trials of panels A-C on separately prepared Au electrodes. Black curves were obtained in dark conditions and blue curves were obtained by irradiating Au electrodes with 2.45 W/cm^2 of 473 nm laser light. A graphite rod was used as the counter electrode.

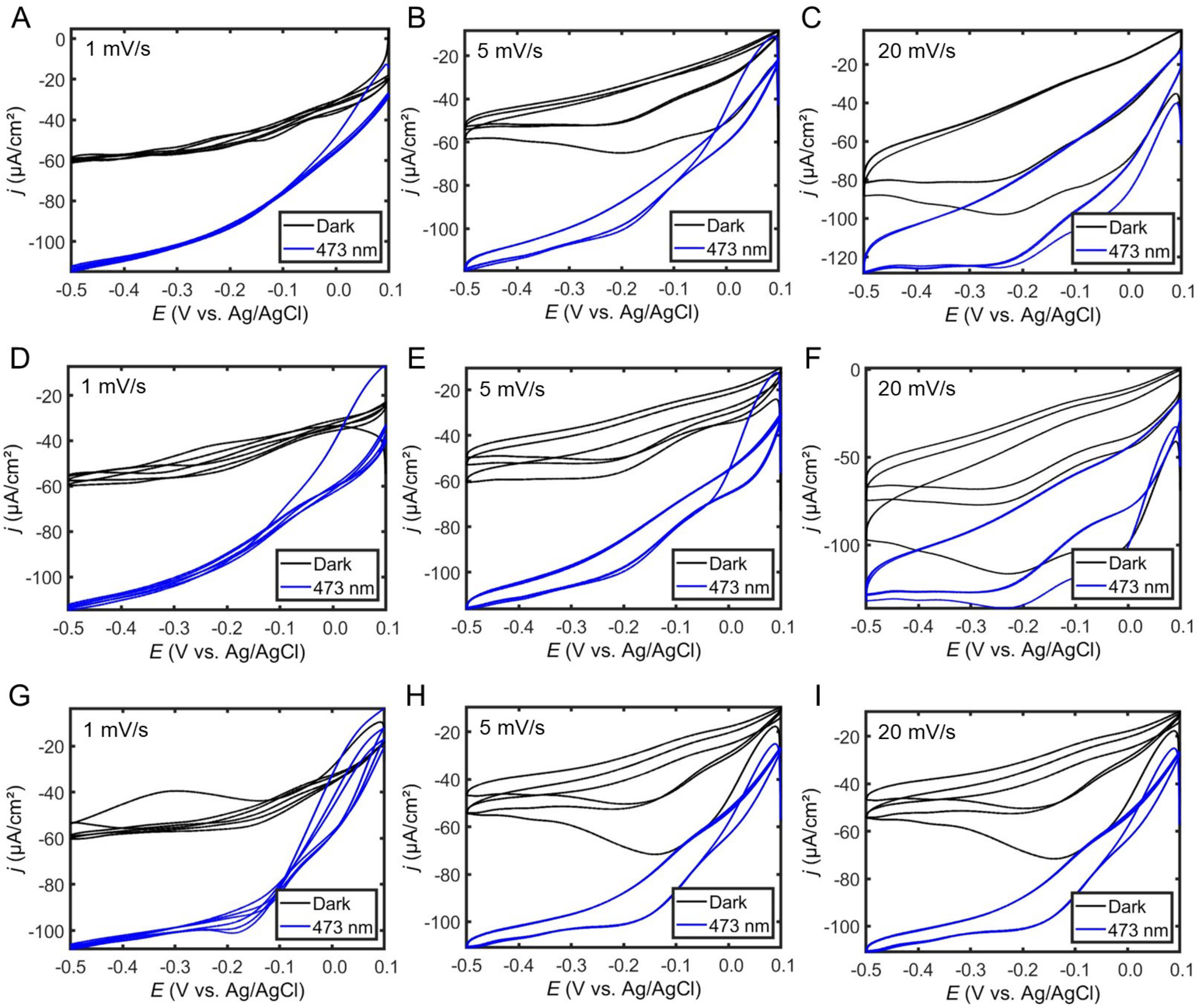

Figure S30. Cyclic voltammograms measured at electrochemically roughened Au working electrodes in an electrolytic solution containing 0.1 M $Na_2HPO_4 \cdot 7H_2O$ (pH = 6.0) with a scan rate of (A) 1 mV/s, (B) 5 mV/s, and (C) 20 mV/s. (D-F) and (G-I) Repeat trials of panels A-C on separately prepared Au electrodes. Black curves were obtained in dark conditions and blue curves were obtained by irradiating Au electrodes with 2.45 W/cm^2 of 473 nm laser light. A graphite rod was used as the counter electrode.

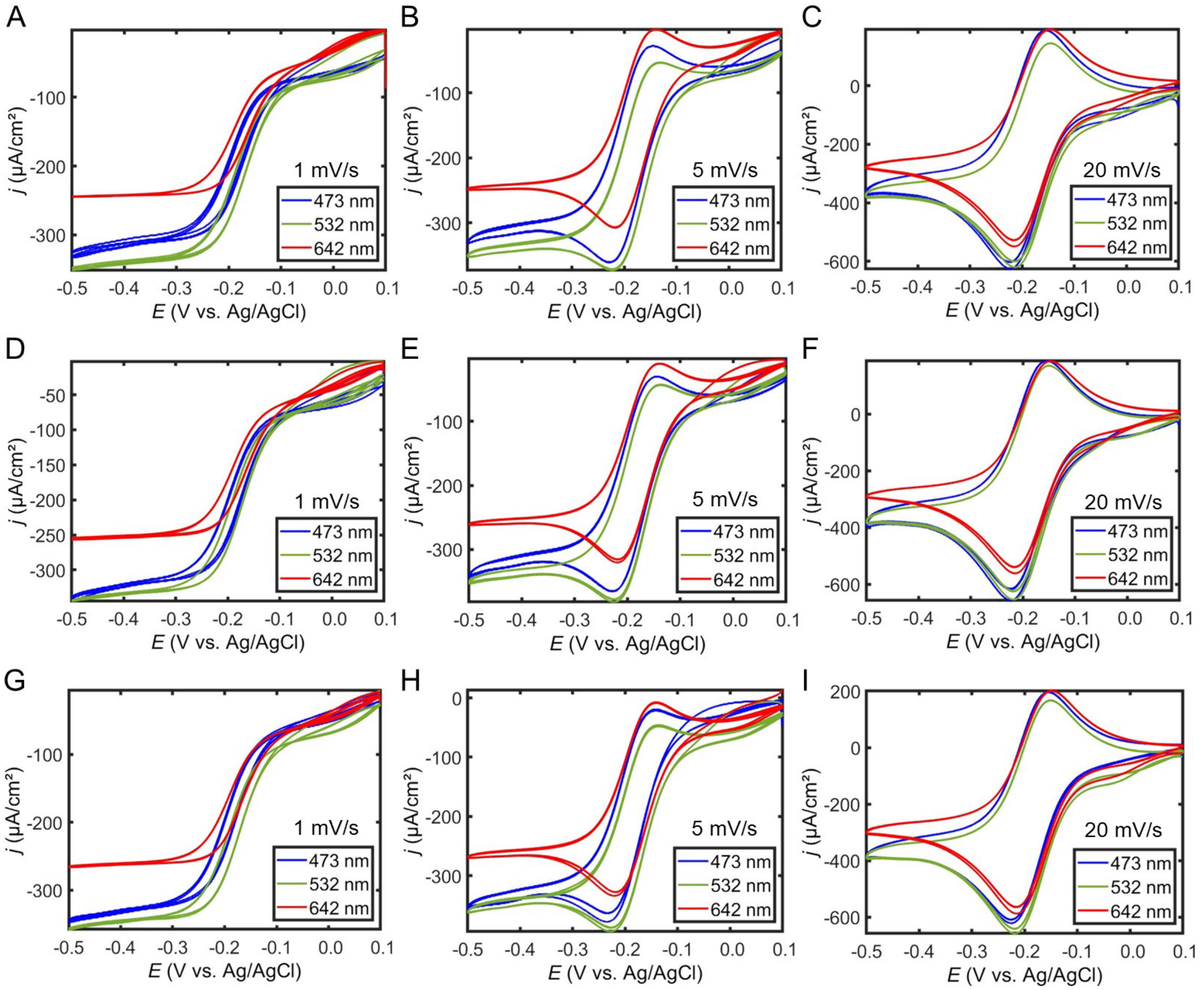

Figure S31. Cyclic voltammograms measured at (A) 1 mV/s, (B) 5 mV/s, and (C) 20 mV/s at electrochemically roughened Au working electrodes in an electrolytic solution containing 0.1 M $Na_2HPO_4 \cdot 7H_2O$ and 5 mM $Ru(NH_3)_6Cl_3 \cdot 6H_2O$ (pH = 6.0) when Au electrodes were irradiated with 2.45 W/cm^2 of 473 nm laser light (blue curves), 2.45 Wcm^2 of 532 nm laser light (green curves), and 2.45 W/cm^2 of 642 nm laser light (red curves). (D-F) and (G-I) are repeat trials of panels A-C on separately prepared Au electrodes. A graphite rod was used as the counter electrode. Data are replotted from Figures S5, S27, and S29.

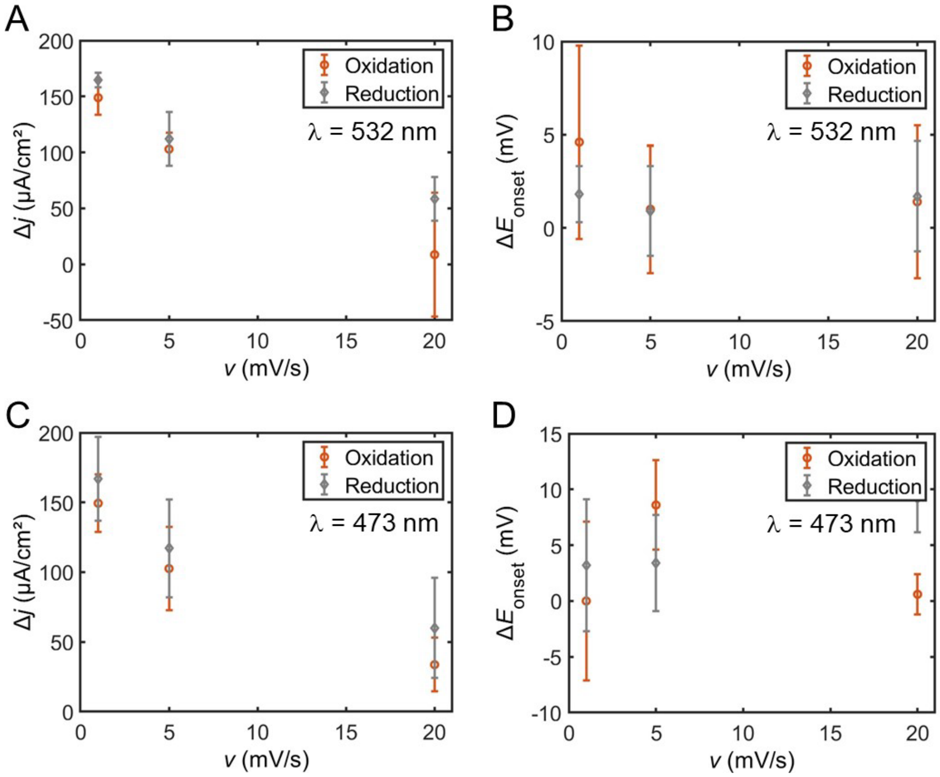

Figure S32. Change in the background subtracted current density (j) and onset potential (E_{onset}) vs. Ag/AgCl (3 M KCl) for the oxidation (orange data) and reduction (gray data) of the $Ru(NH_3)_6^{3+/2+}$ redox probe as a function of scan rate when electrochemically roughened Au electrodes were irradiated with (A, B) 2.45 W/cm^2 of 532 nm laser light and (C, D) 2.45 W/cm^2 of 473 nm laser light. $\Delta E_{onset} = E_{onset,\ light\ irradiation} - E_{onset,\ dark\ conditions}$. $\Delta j = j_{light\ irradiation} - j_{dark\ conditions}$. Oxidation and reduction current densities were tabulated at applied potentials of 0.05 and –0.45 V vs. Ag/AgCl (3 M KCl), respectively. Data points are average values from three independent trials and the errors bars represent the standard deviations of the measurements.

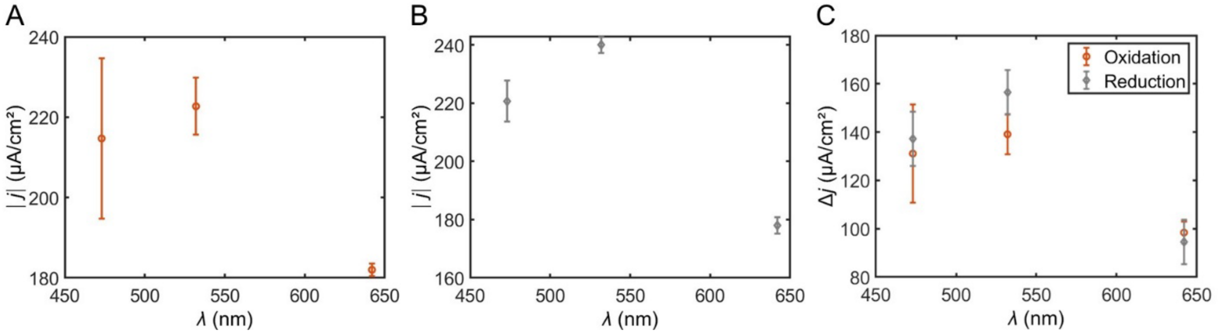

Figure S33. Absolute value of background subtracted current density for the (A) oxidation (orange data) and (B) reduction (gray data) of the $Ru(NH_3)_6^{3+/2+}$ redox probe as a function of excitation wavelength when electrochemically roughened Au electrodes were irradiated with 2.45 W/cm^2 of 642 nm laser light. (C) Change in the background subtracted current density (Δj) for the oxidation (orange data) and reduction (gray data) of the $Ru(NH_3)_6^{3+/2+}$ redox probe as a function of excitation wavelength when electrochemically roughened Au electrodes were irradiated with 2.45 W/cm^2 of 642 nm laser light (j_L) and in dark conditions (j_D). $\Delta j = j_L - j_D$. Scan rate is 1 mV/s. Oxidation and reduction current densities were tabulated at applied potentials of 0.05 and –0.45 V vs. Ag/AgCl (3 M KCl), respectively. Data points are average values from three independent trials and the errors bars represent the standard deviations of the measurements.

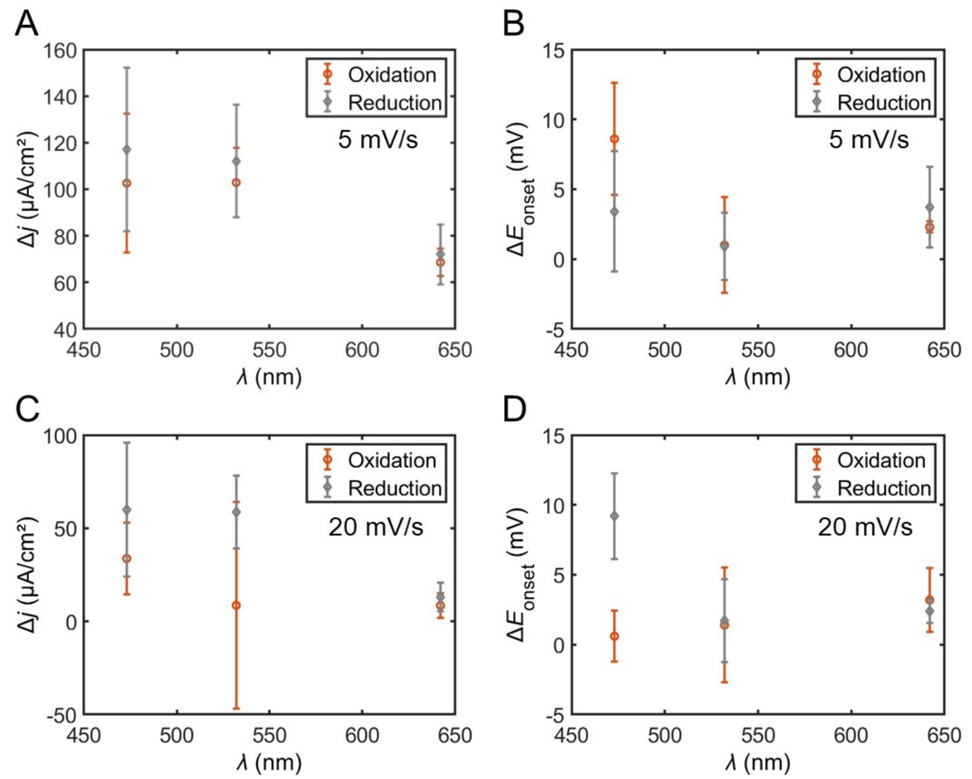

Figure S34. Change in the background subtracted current density (j) and onset potential (E_{onset}) vs. Ag/AgCl (3 M KCl) for the oxidation (orange data) and reduction (gray data) of the $Ru(NH_3)_6^{3+/2+}$ redox probe as a function of excitation wavelength when electrochemically roughened Au electrodes were irradiated with 2.45 W/cm^2 of 473 nm laser light, 2.45 W/cm^2 of 532 nm laser light, and 2.45 W/cm^2 of 642 nm laser light. Data was collected from cyclic voltammograms acquired with a scan rate of (A, B) 5 mV/s and (C, D) 20 mV/s. $\Delta E_{onset} = E_{onset,\ light\ irradiation} - E_{onset,\ dark\ conditions}$. $\Delta j = j_{light\ irradiation} - j_{dark\ conditions}$. Oxidation and reduction current densities were tabulated at applied potentials of 0.05 and –0.45 V vs. Ag/AgCl (3 M KCl), respectively. Data points are average values from three independent trials and the errors bars represent the standard deviations of the measurements.

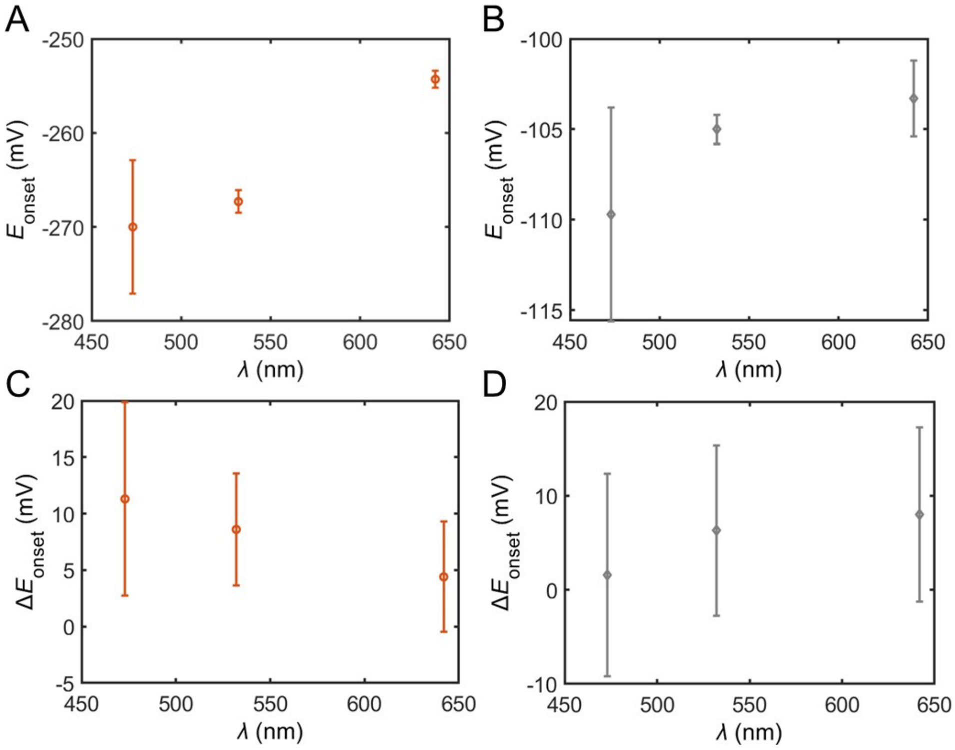

Figure S35. (A, B) Onset potential (E_{onset}) and change in the onset potential (ΔE_{onset}) vs. Ag/AgCl (3 M KCl) for the (A, C) oxidation (orange data) and (B, D) reduction (gray data) of the $Ru(NH_3)_6^{3+/2+}$ redox probe as a function of excitation wavelength when electrochemically roughened Au electrodes were irradiated with 2.45 W/cm^2 of 473 nm laser light, 532 nm laser light, and 642 nm laser light. $\Delta E_{onset} = E_{onset,\ light\ irradiation} - E_{onset,\ dark\ conditions}$. Data points are average values from three independent trials and the errors bars represent the standard deviations of the measurements. Scan rate is 1 mV/s.

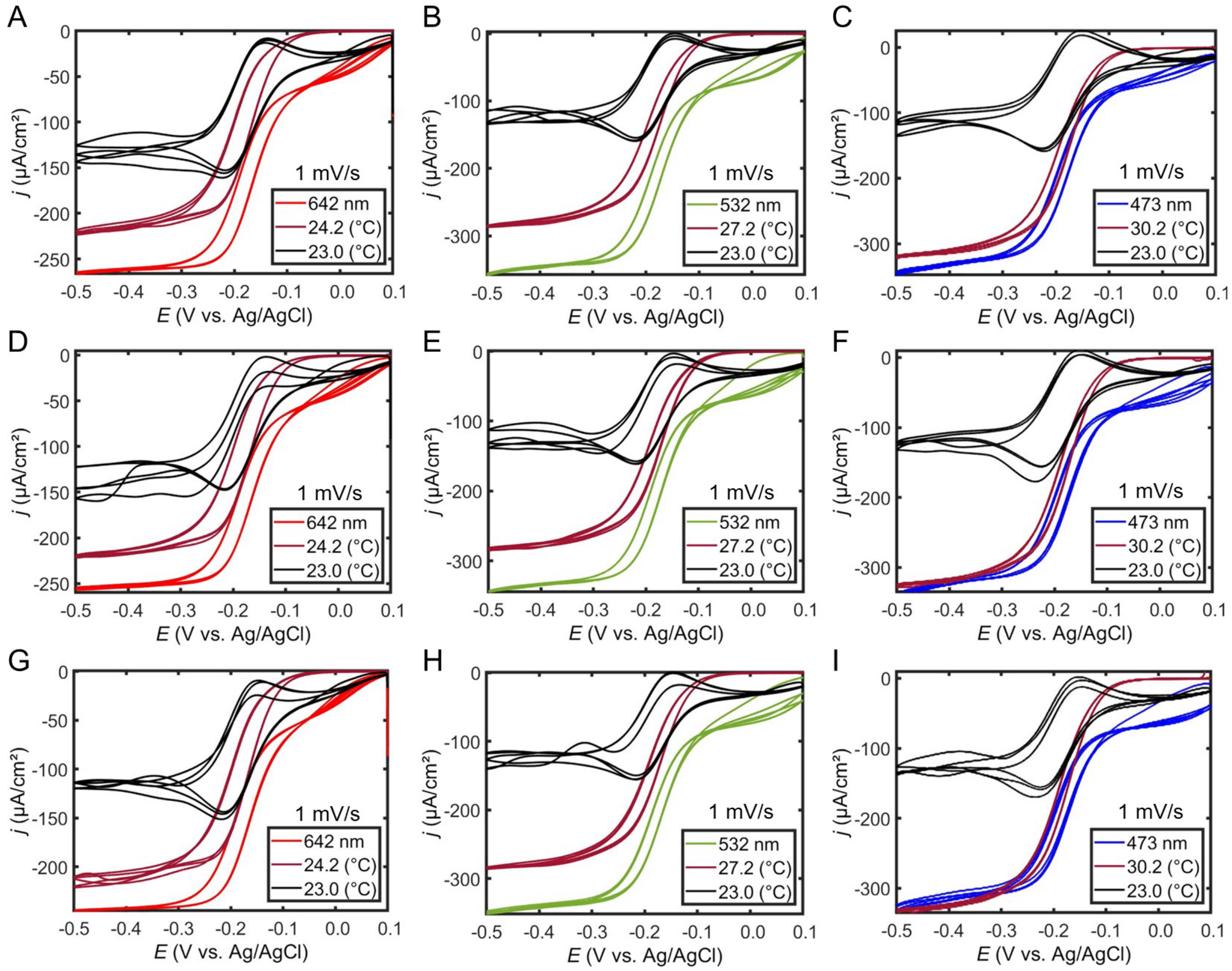

Figure S36. Cyclic voltammograms measured at electrochemically roughened Au working electrodes in an electrolytic solution containing 0.1 M $Na_2HPO_4 \cdot 7H_2O$ and 5 mM $Ru(NH_3)_6Cl_3 \cdot 6H_2O$ (pH = 6.0) at a scan rate of 1 mV/s. Black curves were obtained in dark conditions at room temperature (23.0 °C) and maroon curves were obtained in dark conditions while heating the electrode surface to a temperature equivalent to the surface temperature measured under light irradiation with (A, D, G) 2.45 W/cm^2 of 642 nm laser light, (B, E, H) 2.45 W/cm^2 of 532 nm laser light, and (C, F, I) 2.45 W/cm^2 of 473 nm laser light. Red, green, and blue curves were obtained by irradiating the electrodes with 2.45 W/cm^2 of 642, 532, and 473 nm laser light, respectively. A graphite rod was used as the counter electrode.

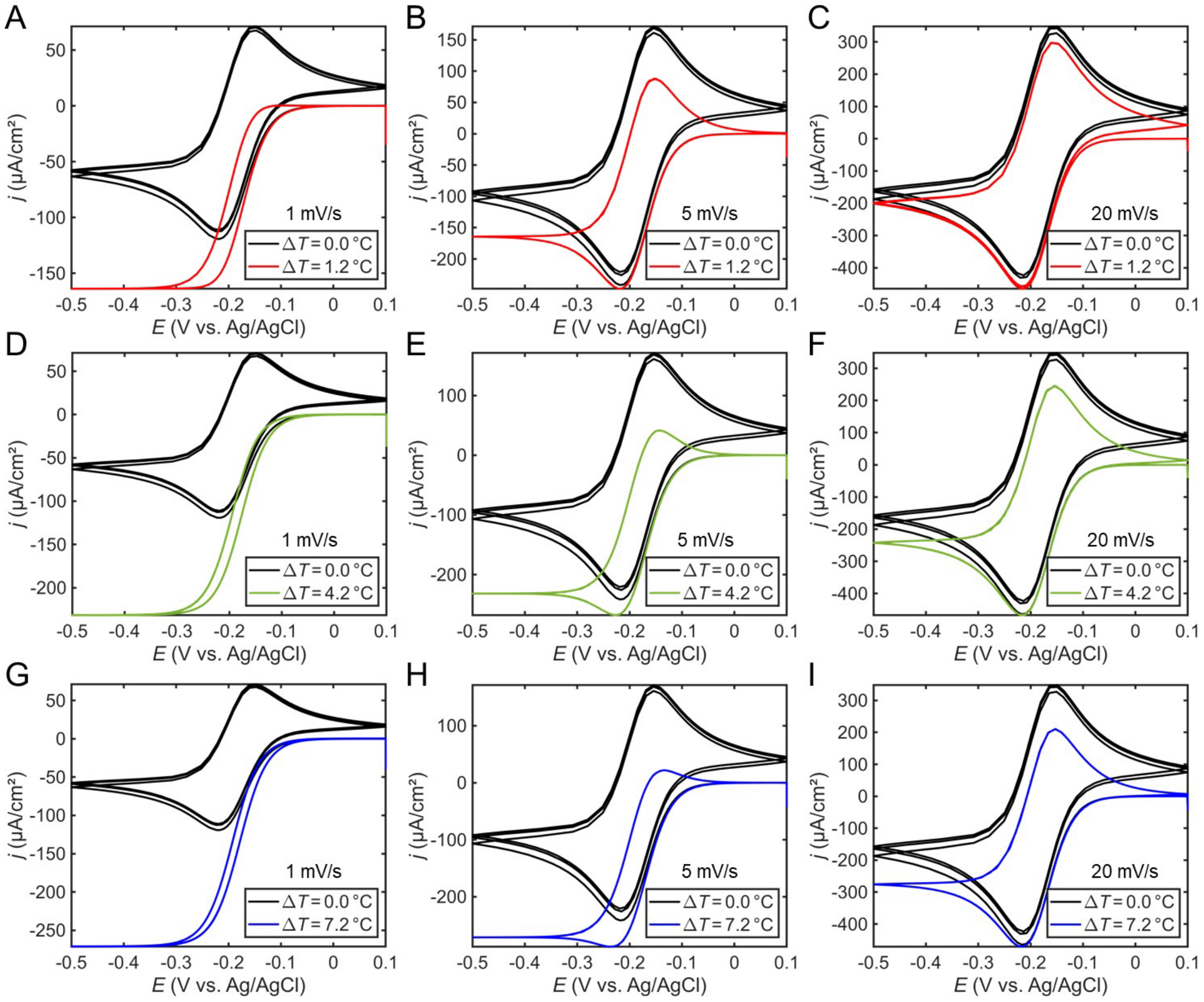

Figure S37. Cyclic voltammograms simulated in an aqueous solvent containing a reversible redox couple with characteristics of $Ru(NH_3)_6^{3+/2+}$ at different scan rates and electrode surface temperatures. All black curves were obtained with the electrode surface and solution temperature set at 23.0 °C (ΔT = 0 °C). Black curves for each scan rate are the same data, reproduced for comparison. (A-C) Red curves were obtained with the electrode surface temperature set at 24.2 °C and the solution temperature set at 23.0 °C (ΔT = 1.2 °C). (D-F) Green curves were obtained with the electrode surface temperature set at 27.2 °C and the solution temperature set at 23.0 °C (ΔT = 4.2 °C). (G-I) Blue curves were obtained with the electrode surface temperature set at 30.2 °C and the solution temperature set at 23.0 °C (ΔT = 7.2 °C). Three potential cycles were simulated for each condition. Panels A and C are reproduced from Figure 6.

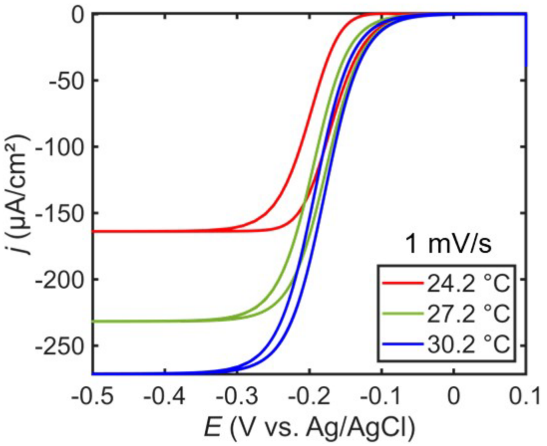

Figure S38. Cyclic voltammograms simulated in an aqueous solvent containing a reversible redox couple with characteristics of $Ru(NH_3)_6^{3+/2+}$ at a scan rate of 1 mV/s and different electrode surface temperatures as indicated in the legend. Red curves were obtained with the electrode surface temperature set at 24.2 °C. Green curves were obtained with the electrode surface temperature set at 27.2 °C. Blue curves were obtained with the electrode surface temperature set at 30.2 °C. In each condition, the solution temperature is set at 23.0 °C. Three potential cycles were simulated for each condition. Curves are reproduced from panels A, D, and G from Figure S37 and overlaid here for comparison.

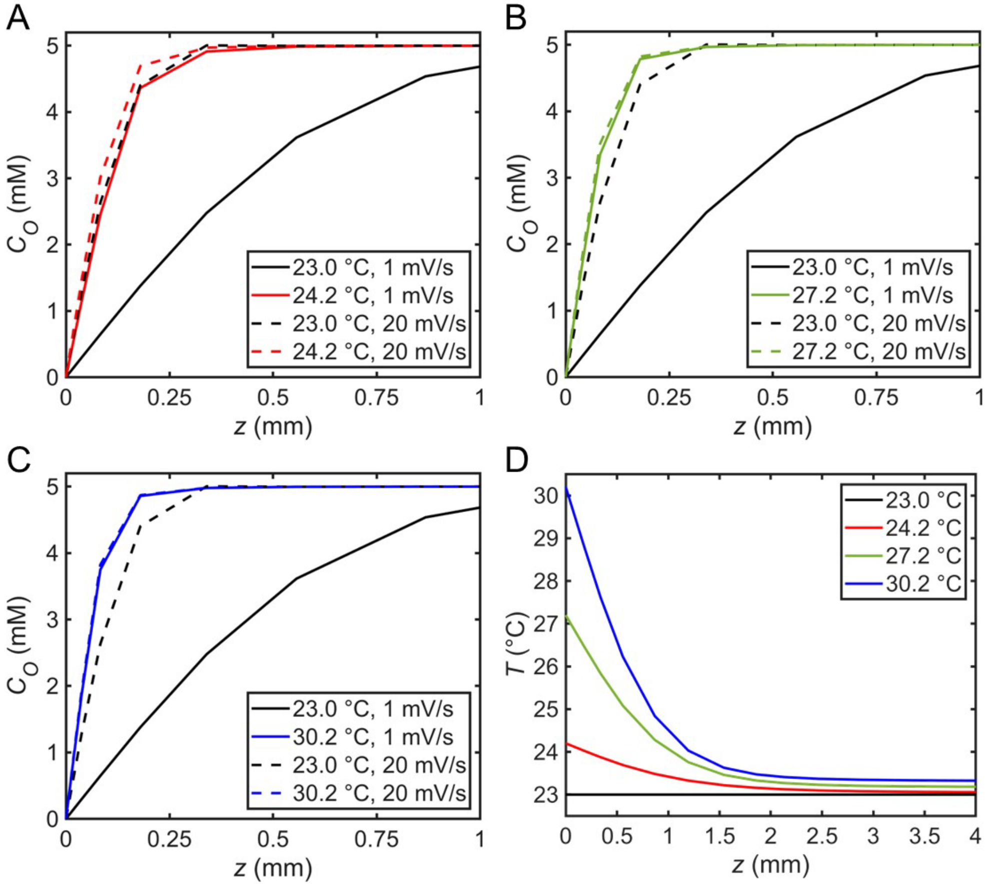

Figure S39. One-dimensional (A-C) concentration and (D) temperature gradients obtained from simulations of $Ru(NH_3)_6^{3+}$ electrochemical reduction at steady-state conditions. The electrode surface temperature is set at (A) 24.2 °C (red curves), (B) 27.2 °C (green curves), and (C) 30.2 °C (blue curves). In all panels, black curves were obtained with the electrode surface temperature set at 23.0 °C. The aqueous solvent temperature is set to an initial temperature of 23.0 °C. Concentration gradients are shown for scan rates of 1 mV/s (solid lines) and 20 mV/s (dashed lines). Black curves in panels A-C are the same data, reproduced for comparison.

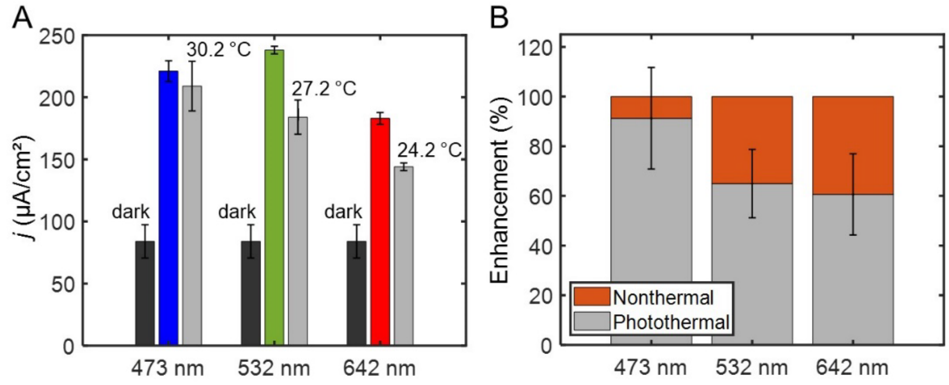

Figure S40. (A) $Ru(NH_3)_6^{3+}$ reduction current densities tabulated at -0.45 V vs. Ag/AgCl from background subtracted cyclic voltammograms acquired at electrochemically roughened Au disk electrodes. Dark gray bars are data collected in dark conditions, colored bars are data collected when irradiating the plasmonic working electrode with 2.45 W/cm^2 of 473 nm (blue), 532 nm (green), or 642 nm (red) laser light. Light gray bars are data collected in dark conditions with the working electrode temperature set at 30.2 °C, 27.2 °C, or 24.2 °C, corresponding to the working electrode temperature measured when irradiated with the respective wavelengths on light. Data points are average values from three independent trials and the errors bars represent the standard deviations of the measurements. (B) Contributions of photothermal heating (light gray) and nonthermal effects (orange) to the enhanced reduction current densities when the plasmonic electrodes are irradiated with light.
